# Age-Related Hyperphosphatemia Is Associated with Metabolic and Mitochondrial Alterations During Myogenic Differentiation and in Skeletal Muscle from Old Mice

**DOI:** 10.3390/ijms27135662

**Published:** 2026-06-23

**Authors:** María Martos-Elvira, Alberto Guerrero-Méndez, Ariadna Moreno-Piedra, Javier Sanz-Zamora, Elena Alcalde-Estévez, Marta Ruiz-Ortega, Natalia Carrillo-López, Susana López-Ongil, Gemma Olmos, María Piedad Ruiz-Torres

**Affiliations:** 1 Universidad de Alcalá, Facultad de Medicina y Ciencias de la Salud, Departamento de Biología de Sistemas, Alcalá de Henares, 28871 Madrid, Spain; maria.martos@uah.es (M.M.-E.); alberto.guerreromend@uah.es (A.G.-M.); javier.sanzz@uah.es (J.S.-Z.); elena.alcaldee@uah.es (E.A.-E.); gemma.olmos@uah.es (G.O.); mpiedad.ruiz@uah.es (M.P.R.-T.); 2 Universidad de Alcalá, Instituto Ramón y Cajal de Investigación Sanitaria (IRYCIS), 28034 Madrid, Spain; ariadna.moreno@uah.es; 3 Área 5-Fisiología y Fisiopatología Renal y Vascular del IRYCIS, 28034 Madrid, Spain; 4 Redes de Investigación Cooperativa Orientadas a Resultados en Salud (RICORS), RICORS2040-Renal, 28029 Madrid, Spain; marta.ruiz.ortega@uam.es (M.R.-O.);; 5 Laboratorio de Biología Molecular y Celular en Patología Renal y Vascular, Departamento de Medicina, IIS-Fundación Jiménez Díaz, Universidad Autónoma de Madrid, 28040 Madrid, Spain; 6 Unidad de Metabolismo Óseo, Unidad de Gestión Clínica de Medicina Interna, Metabolismo Óseo, Vascular y Enfermedades Inflamatorias Crónicas, Instituto de Investigación Sanitaria del Principado de Asturias (ISPA), Hospital Universitario Central de Asturias (HUCA), 33011 Oviedo, Spain; 7 Unidad de Investigación de la Fundación para la Investigación Biomédica, Hospital Universitario Príncipe de Asturias, Alcalá de Henares, 28805 Madrid, Spain; 8 Instituto Reina Sofía de Investigación Nefrológica (IRSIN), Fundación Renal Iñigo Álvarez de Toledo (FRIAT), 28003 Madrid, Spain

**Keywords:** sarcopenia, hyperphosphatemia, mitochondria, metabolic switch

## Abstract

Age-related hyperphosphatemia is increasingly recognized as a contributing factor in sarcopenia. This work studies the metabolic effects of elevated phosphate on muscle. C2C12 cells were differentiated in the absence or presence of 10 mM β-glycerophosphate (BGP), an exogenous phosphate donor. In addition, quadriceps muscles from four experimental groups of male C57BL/6J mice were analyzed: young (5 months) and old (24 months) fed with standard diet; old mice fed with hypophosphatemic diet or supplemented with the phosphate binder Velphoro^®^, for the last three months of life. Mice were stratified according to sarcopenia degree based on muscle mass, strength and physical performance. Protein levels were determined by immunoblotting and mRNA expression by RT-qPCR. ATP levels were measured by luminescence and L-lactate production, citrate synthase and cytochrome c oxidase activities by colorimetric assays. Mitochondrial content, membrane potential and reactive oxygen species (ROS) were determined by fluorescence assay. BGP-treated cells showed increased glucose transporter 1 (GLUT1) and decreased NADH Dehydrogenase (CI-NDUFB8) protein expression, elevated hexokinase II (HK2), phosphoglycerate kinase 1 (PGK1) and lactate dehydrogenase A (LDHA) mRNA levels, reduced ATP levels, increased lactate production, and decreased mitochondrial enzyme activities. Moreover, BGP increased ROS, diminished mitochondrial membrane potential, and altered fusion–fission dynamics and mitophagy. In aged quadriceps, oxidative phosphorylation (OXPHOS) subunits and superoxide dismutase 2 (SOD2) expression were reduced. The hypophosphatemic diet improved all parameters, whereas Velphoro^®^ selectively increased Mitochondrial cytochrome C oxidase subunit 1 (CIV-MTCO1) expression. Several altered mitochondrial markers are associated with sarcopenia degree. Altogether, hyperphosphatemia induces metabolic changes that scale with the sarcopenic degree. Our findings show a relevant association between hyperphosphatemia and mitochondrial dysfunction, and they support the potential benefit of phosphate reduction as a strategy to prevent or mitigate sarcopenia.

## 1. Introduction

Ageing contributes to the development of numerous chronic diseases that affect multiple tissues, including skeletal muscle [1]. Age-related changes in skeletal muscle lead to various structural and functional alterations, which in turn impair performance and increase physical limitations [2]. One of the most clinically relevant ageing-related conditions is sarcopenia, defined as the progressive loss of muscle mass and function, leading to reduced strength and greater frailty [3]. Sarcopenia, therefore, represents a condition of significant clinical importance, with consequences such as impaired mobility, increased risk of falls and fractures, loss of independence, and higher mortality [4]. Although several pathways and mechanisms contributing to sarcopenia have been described, the underlying precise processes remain incompletely understood. Different factors can contribute to ageing-related pathologies, including telomere loss, genomic instability, chronic inflammation, impaired protein homeostasis, defective macroautophagy, dysregulated nutrient sensing, and cellular senescence [5]. Among these, mitochondrial dysfunction plays a central role in skeletal muscle ageing, because when mitochondrial quality control and bioenergetics capacity are impaired, muscle homeostasis is compromised and functional decline occurs [6]. Mitochondrial metabolism is particularly important in skeletal muscle, as contraction during physical activity is highly energy-demanding and requires substantial ATP production [7]. Ageing reduces the activity of electron transport chain complexes, leading to lower ATP synthesis, as well as diminished mitochondrial enzymatic activity and increased reactive oxygen species production [8,9]. Collectively, these changes may cause metabolic insufficiency in ageing muscle, potentially contributing to progressive reductions in muscle function [10]. The observed deterioration in muscle mass and performance is thought to result, at least in part, from the metabolic and cellular alterations that accompany age-related structural and functional changes in skeletal muscle. On the other hand, an important role has been described for hyperphosphatemia, the ageing-related increase in the serum phosphate concentration associated with deregulation of parathyroid hormone (PTH), vitamin D, Klotho, and FGF23 [11], in the appearance of sarcopenia signs.

Previous results from our laboratory have shown that hyperphosphatemia induces senescence in cultured myoblasts from mice [12] and compromises myogenic differentiation of cultured myoblasts by inhibiting MyoD transcriptional activity, decreasing expression of myogenic factors and increasing expression of profibrotics factors [13]. Moreover, structural and functional analyses of skeletal muscle in aged mice revealed a strong association between hyperphosphatemia and sarcopenic features, including reduced muscle mass, altered fiber composition, fibrosis, and age-related changes in muscle function [14]. However, the exact mechanisms by which hyperphosphatemia contribute to sarcopenia have not been fully elucidated.

During the process of myogenic differentiation from myoblasts to mature myotubes, skeletal muscle cells undergo profound metabolic remodeling, transitioning from the predominantly glycolytic profile characteristic of proliferating myoblasts to the oxidative metabolic phenotype of differentiated myotubes [15]. Despite the central role of metabolic regulation in muscle physiology, no studies have yet clarified how hyperphosphatemia affects the bioenergetic profile or mitochondrial function of skeletal muscle cells in the context of sarcopenia. Given that hyperphosphatemia has been shown to impair myogenic differentiation, we hypothesized that this defect may arise from phosphate-induced disruptions in the metabolic reprogramming required for the shift from myoblast proliferation to terminal differentiation.

Based on these considerations, the aim of the present study was to characterize the metabolic adaptations occurring during myogenic differentiation under hyperphosphatemic conditions and to compare them with those observed under standard differentiation conditions. To this end, we have employed a mouse myoblast cell line differentiated for seven days in the presence of horse serum, a widely established in vitro model for investigating the metabolic and molecular mechanisms driving myogenic differentiation [16]. Furthermore, to evaluate the physiological relevance of our in vitro findings, complementary experiments have been performed in an aged mouse model using 24-month-old animals. These mice have been fed, for three months, either a low-phosphate diet or supplemented with Velphoro^®^, a phosphate binder, to reduce serum phosphate levels. The expression of key metabolic enzymes has been analyzed in skeletal muscle from these mice and compare with age-matched animals maintained on a standard diet. Finally, we have assessed whether the expression patterns of these enzymes correlate with the severity of sarcopenia, thereby providing insight into the metabolic pathways contributing to muscle decline in ageing.

Collectively, these studies may offer critical advances in understanding the metabolic mechanisms underlying sarcopenia and highlight the potential of modulating serum phosphate levels as a strategy for its prevention or attenuation.

## 2. Results

### 2.1. Hyperphosphatemia Alters Cellular Metabolism and Reduces ATP Levels During C2C12 Cell Differentiation

**Figure 1:**
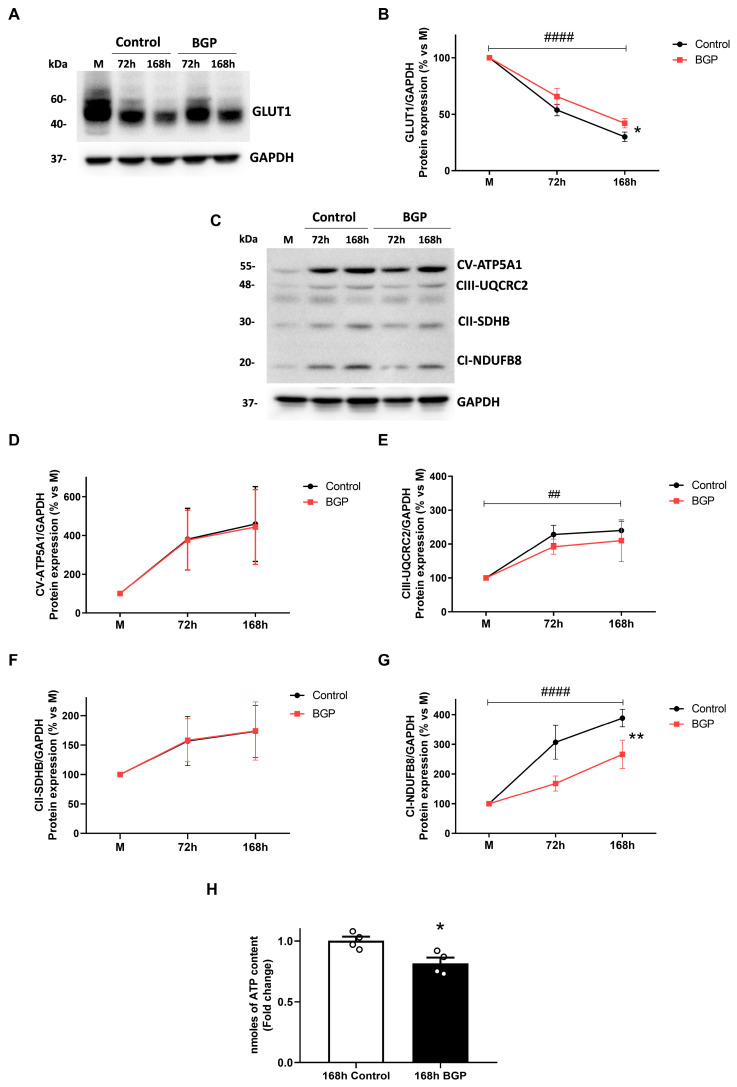
Hyperphosphatemia impairs cellular metabolism in differentiating C2C12 cells. C2C12 cells were analyzed at undifferentiated myoblasts (M), 72 h and 168 h of differentiation in theabsence (control) or presence of 10 mM β-glycerophosphate (BGP) during differentiation. (**A**) GLUT1 protein expression was evaluated by Western blot. (**B**) Graph represents the densitometric analysis of the bands. (**C**) Oxidative phosphorylation (OXPHOS)-related enzymes protein expression was evaluated by Western blot. Graphs represent densitometric analysis of protein expressions of (**D**) CV-ATP5A1, (**E**) CIII-UQCRC2, (**F**) CII-SDHB and (**G**) CI-NDUFB8 subunits. Representative images of each one are shown. Densitometries were normalized to endogenous GAPDH. The results are expressed as percentage of M and are the mean ± standard error of the mean (SEM) from 8 (GLUT1) or 4 (OXPHOS-related enzymes) different experiments. Significant main effects are shown for treatment (* *p* < 0.05; ** *p* < 0.01) and time (## *p* < 0.01; #### *p* < 0.0001). (**H**) ATP nmoles were measured by luminescence assay at 168 h of differentiation. Results are expressed as individual data points relative to the mean of 168 h control (* *p* < 0.05) and are presented as the mean ± SEM from 4 independent experiments.

We first analyzed whether hyperphosphatemia affects cellular metabolism during the myogenic differentiation process in C2C12 cells grown with 2% horse serum (HS). Under control conditions, the expression of glucose transporter 1 (GLUT1, a key protein of glycolytic metabolism) progressively decreases as the cells differentiate (Figure 1A,B (#### *p* < 0.0001)). In contrast, the protein expression of electron transport chain (ETC) subunits, assessed by the content of ATP Synthase (CV-ATP5A1), Coenzyme Q (CIII-UQCRC2), Succinate Dehydrogenase (CII-SDHB), and NADH Dehydrogenase (CI-NDUFB8), progressively increases during differentiation (Figure 1C–E (## *p* = 0.0014); Figure 1F,G (#### *p* < 0.0001)). Treatment of C2C12 cells with 10 mM of β-glycerophosphate (BGP), an exogenous phosphate donor, during myogenic differentiation for 168 h, resulted in a higher expression of GLUT1 (* *p* = 0.0304) (Figure 1A,B) compared with cells differentiated under control conditions, together with a significant decrease (** *p* = 0.0062) in the expression of the catalytic subunit CI-NDUFB8 (Figure 1G). No changes were observed in the other ETC subunits analyzed (Figure 1C–F). In some experiments, we have verified that BGP reduced myogenic differentiation by evaluating Myosin Heavy Chain (MHC) protein expression by immunoblot, as previously reported [12]. (See Supplementary Figure S1). In addition, ATP production measured in 168 h myotubes showed a significant decrease (*p* = 0.0374) in cells treated with BGP during the differentiation process (Figure 1H). Altogether, these results suggest that elevated extracellular phosphate induces a metabolic shift during myogenic differentiation.

### 2.2. Hyperphosphatemia Increases Glycolytic Metabolism and Lactate Production in Differentiated C2C12 Cells

**Figure 2:**
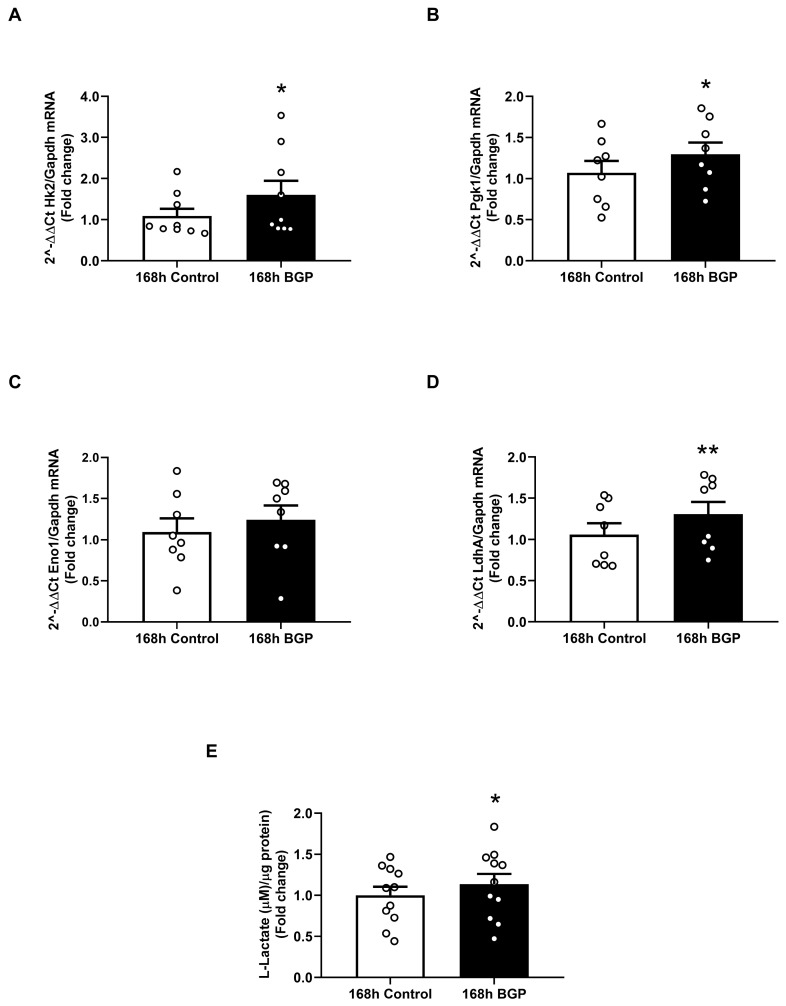
Hyperphosphatemia increases the expression of key proteins and genes of glycolytic metabolism and lactate production in differentiated C2C12 cells. All the analyses were performed in myotubes with 168 h of differentiation in the absence (control) or presence of 10 mM BGP during the differentiation process. mRNA expressions of (**A**) *Hk2*, (**B**) *Pgk1*, (**C**) *Eno1* and (**D**) *LdhA* were measured using RT-qPCR. Relative fold change values were normalized against *Gapdh* as endogenous control. Results are expressed as individual data points relative to the mean of 168 h control (* *p* < 0.05; ** *p* < 0.01) and are presented as the mean ± SEM from 9 (*Hk2*) or 8 (*Pgk1*, *Eno1* and *LdhA*) different experiments. (**E**) Measurement of extracellular L-lactate using a fluorimetric assay. Results are expressed as individual data points relative to the mean of 168 h control (* *p* < 0.05) and are presented as the mean ± SEM from 11 independent experiments.

Considering the shift towards a more glycolytic pattern in cells treated with BGP (10 mM) during myogenic differentiation, various genes involved in glycolysis were studied in mature myotubes. Cells differentiated for 168 h in the presence of BGP presented a significant increase in mRNA expression of hexokinase II (*Hk*2) (*p* = 0.0443), phosphoglycerate kinase 1 (*Pgk1*) (*p* = 0.0232) and lactate dehydrogenase A (*LdhA*) (*p* = 0.0078) compared with control cells (Figure 2A, Figure 2B and Figure 2D, respectively). In contrast, Enolase-1 (*Eno1)* did not present significant changes (Figure 2C). In addition, levels of the main product of glycolytic metabolism, L-lactate, were measured revealing a significant increase (*p* = 0.0249) in BGP-treated C2C12 myotubes (Figure 2E). These findings reinforce the role that high phosphate levels play inducing a transition towards elevated glycolytic metabolism in skeletal muscle cells.

### 2.3. Hyperphosphatemia Impairs Mitochondrial Function and Increases Oxidative Stress in Mature Myotubes

**Figure 3:**
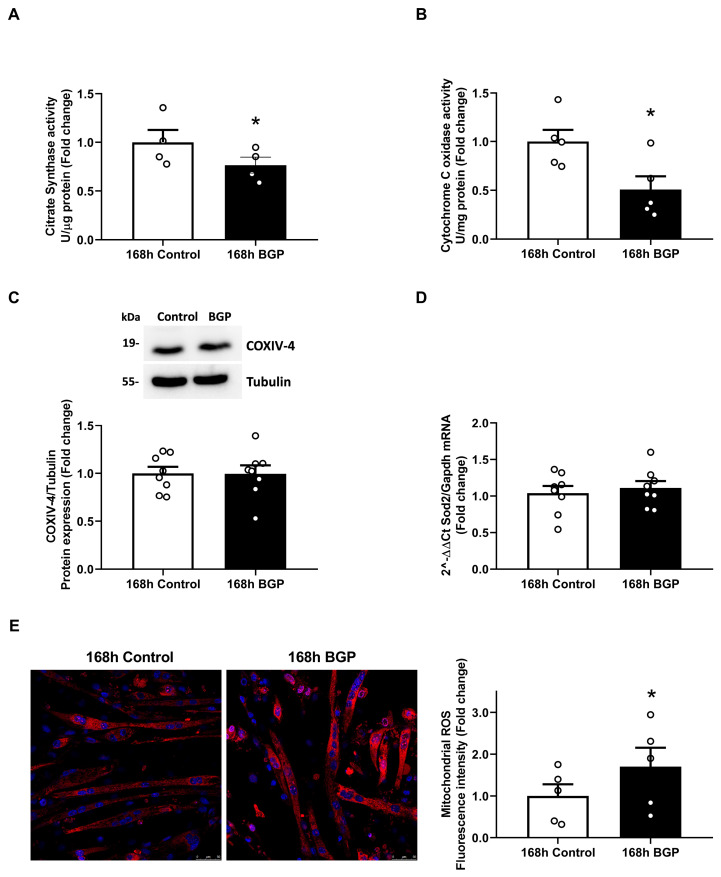
Hyperphosphatemia induces alterations in mitochondrial function and increases mitochondrial reactive oxygen species (ROS) in differentiated C2C12 cells. These studies were performed in differentiated cells for 168 h in the absence (control) or presence of 10 mM BGP during the differentiation process. (**A**) Citrate synthase activity was measured by colorimetric assay. Results are expressed as individual data points relative to the mean of 168 h control (* *p* < 0.05) and are presented as the mean ± SEM from 4 independent experiments. (**B**) Activity of cytochrome C oxidase was measured by colorimetric assay. Results are expressed as individual data points relative to the mean of 168 h control (* *p* < 0.05) and are presented as the mean ± SEM from 5 different experiments. (**C**) Protein expression of COXIV-4 was studied by Western blot. A representative blot is shown. Bar graph represents the densitometric analysis of the bands. Densitometries were normalized to endogenous Tubulin. Results are expressed as individual data points relative to the mean of 168 h control and are presented as the mean ± SEM from 8 different experiments. (**D**) mRNA expression of superoxide dismutase 2 (*Sod2*) was analyzed by RT-qPCR. Relative fold change values were normalized against *Gapdh* as endogenous control and expressed as individual data points relative to the mean of 168 h control. They are the mean ± SEM from 8 different experiments. (**E**) Mitochondrial ROS production was determined by using superoxide mitochondrial probe (MitoSOX^TM^, red fluorescence) and normalized by nuclei of myotubes content (Hoechst in blue). In vivo cells were visualized by confocal microscopy. Representative pictures obtained with 40× magnification are shown. Scale bars: 50µM. Bar graph represents results as individual data points relative to the mean of 168 h control (* *p* < 0.05) of fluorescence intensity. Results are the mean ± SEM from 5 different experiments.

To explore the mechanism involved in this shift towards glycolytic metabolism in cells treated with BGP during differentiation, mitochondrial function was studied. First, we measured the citrate synthase activity, a key maker of mitochondrial function. Cells treated with BGP (10 mM) during differentiation for 168 h showed a decrease in citrate synthase activity (*p* = 0.0271) compared to control differentiated cells (Figure 3A). Furthermore, to confirm whether high phosphate induced a dysregulation of oxidative phosphorylation, cytochrome C oxidase activity was measured. A significant decrease was observed in differentiated treated cells (*p* = 0.0479) (Figure 3B), despite no significant changes being found in subunit 4 of the cytochrome C complex (COXIV-4) protein expression (Figure 3C). On the other hand, when mitochondria are damaged, a redox imbalance occurs and increases oxidative stress. No changes were found in the mRNA expression of one of the main enzymes regulating mitochondrial oxidative stress, superoxide dismutase 2 (*Sod2*) (Figure 3D). However, mitochondrial reactive oxygen species (ROS) were analyzed using a mitochondrial superoxide indicator (MitoSOX^TM^) by confocal microscopy, showing an increase in oxidative stress after treatment with BGP for 168 h during differentiation (*p* = 0.0401) (Figure 3E). Thus, when this ROS increase was reduced with the ROS scavenger, N-acetylcysteine (NAC), the protein expression of GLUT1 decreased, and mRNA expression of *Hk2* did not change, in cells treated with NAC and BGP compared with cells only treated with BGP during myogenic differentiation (Supplementary Figure S2). All these results point to mitochondrial dysfunction during the myogenic differentiation process induced by high levels of phosphate.

### 2.4. Hyperphosphatemia Disrupts Mitochondrial Health and Dynamics in Differentiated C2C12 Cells

**Figure 4:**
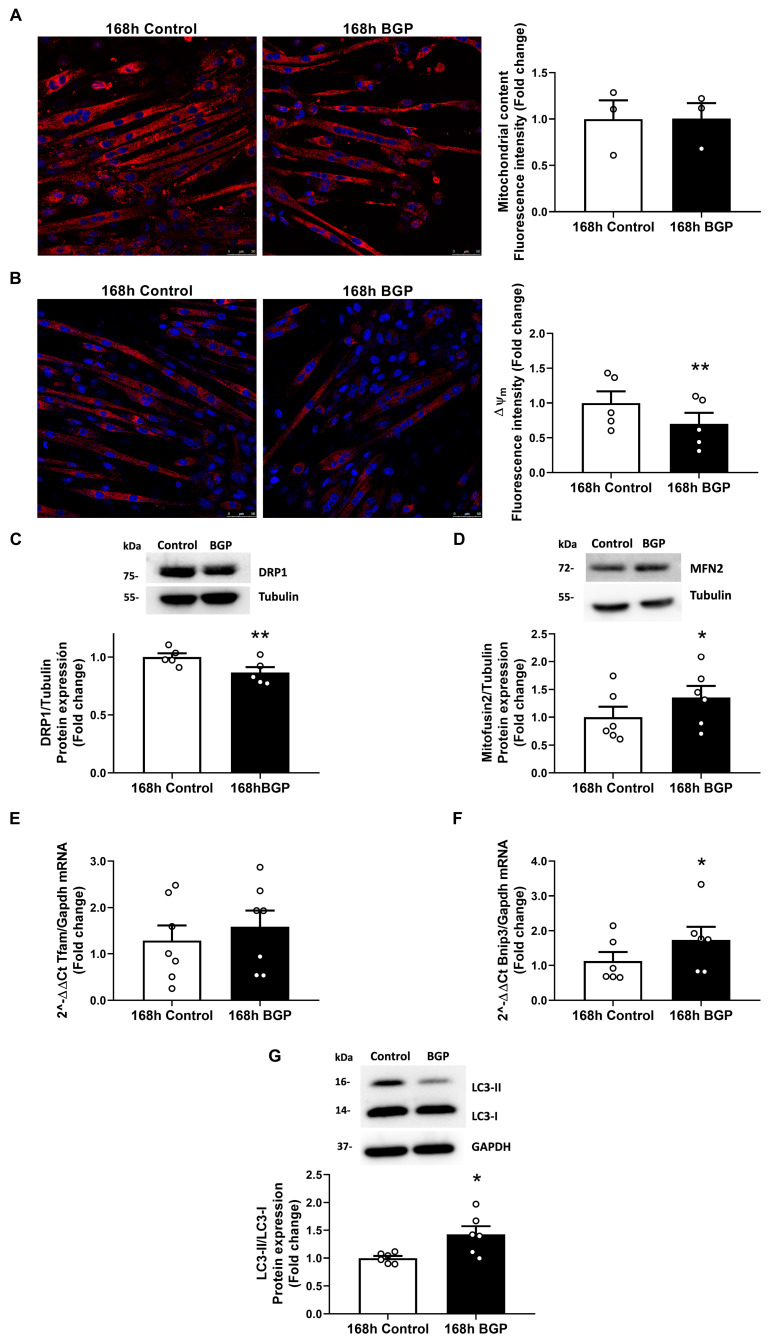
Hyperphosphatemia impairs mitochondrial health and dynamics in C2C12 myotubes. Cells were differentiated for 168 h in the absence (control) or presence of 10 mM BGP during the differentiation process. (**A**) Mitochondrial content and (**B**) mitochondrial membrane potential were observed using the fluorescent probes MitoTracker^TM^ and TMRM, respectively (red fluorescence). Results were normalized by nuclei of myotubes (Hoechst in blue). Representative confocal microscopy images with 40× magnification are shown. Scale bars: 50µM. Bar graph show results as individual data points relative to the mean of 168 h control (** *p* < 0.01) of fluorescence intensity. Results are the mean ± SEM from 3 and 5 different experiments respectively. (**C**) DRP1 and (**D**) MFN2 protein expressions were determined by Western blot. Representative blots are shown. Bar graphs represent densitometric analysis of the bands. Densitometries were normalized to endogenous Tubulin. The results are expressed as individual data points relative to the mean of 168 h control (* *p* < 0.05; ** *p* < 0.01) and are the mean ± SEM from 5 and 6 different experiments, respectively. (**E**) *Tfam* and (**F**) *Bnip3* mRNA expressions were analyzed by RT-qPCR. Relative fold change values were normalized against *Gapdh* as endogenous control and expressed as individual data points relative to the mean of 168 h control (* *p* < 0.05). Results are the mean ± SEM from 7 and 6 different experiments respectively. (**G**) Ratio LCB3-II/LCB3-I was measured by Western blot. Representative blot is shown. Graph represents the densitometric analysis of the rate LC3B isoform II/isoform I. The results are expressed as individual data points relative to the mean of 168 h control (* *p* < 0.05) and are the mean ± SEM from 6 different experiments.

To further explore the mechanisms underlying the effect of hyperphosphatemia on mitochondria, we studied some other parameters related to mitochondrial health. To analyze the effect of hyperphosphatemia on mitochondrial viability, mitochondrial content was measured using the fluorescent dye MitoTracker^TM^ by confocal microscopy and did not show significant changes in differentiated myotubes in the presence of BGP (Figure 4A). In addition, to determine whether mitochondria were damaged, mitochondrial membrane potential (ΔΨm) was measured with a Tetramethylrhodamine methyl ester (TMRM) fluorescence probe by confocal microscopy, which accumulates in intact mitochondria. BGP produces a significant decrease in TMRM compared to cells under control conditions during myogenic differentiation (*p* = 0.0064) (Figure 4B).

On the other hand, some mitochondrial dynamics markers were studied. Results showed a significant decrease in the fission Dynamin-1-like protein (DRP1) (*p* = 0.0014) and an increase in fusion protein Mitofusin 2 (MFN2) (*p* = 0.0283) in cells differentiated under BGP treatment (Figure 4C,D). Furthermore, mRNA expression of mitochondrial transcription factor (*Tfam*), which is related with mitochondrial biogenesis, showed a growing trend (Figure 4E). Another sign of mitochondrial damage is enhanced mitophagic flux. To test this, some mitophagy markers were studied. BGP induced an upregulation in mRNA expression of mitophagy factor BCL2/adenovirus E1B 19 kDa protein-interacting protein 3 (*Bnip3*) (*p* = 0.0397) (Figure 4F). In addition, there was an increase in protein expression of LC3-II/LC3-I ratio in treated cells (*p* = 0.0344) (Figure 4G). Taken together, these results indicate that high phosphate levels cause mitochondrial dysregulation, leading to a decrease in mitochondrial function, an imbalance in mitochondrial dynamics and an increase in markers of mitophagy.

### 2.5. Aged Mice Present Mitochondrial Alterations in Skeletal Muscle Without Glycolytic Compensation

**Figure 5:**
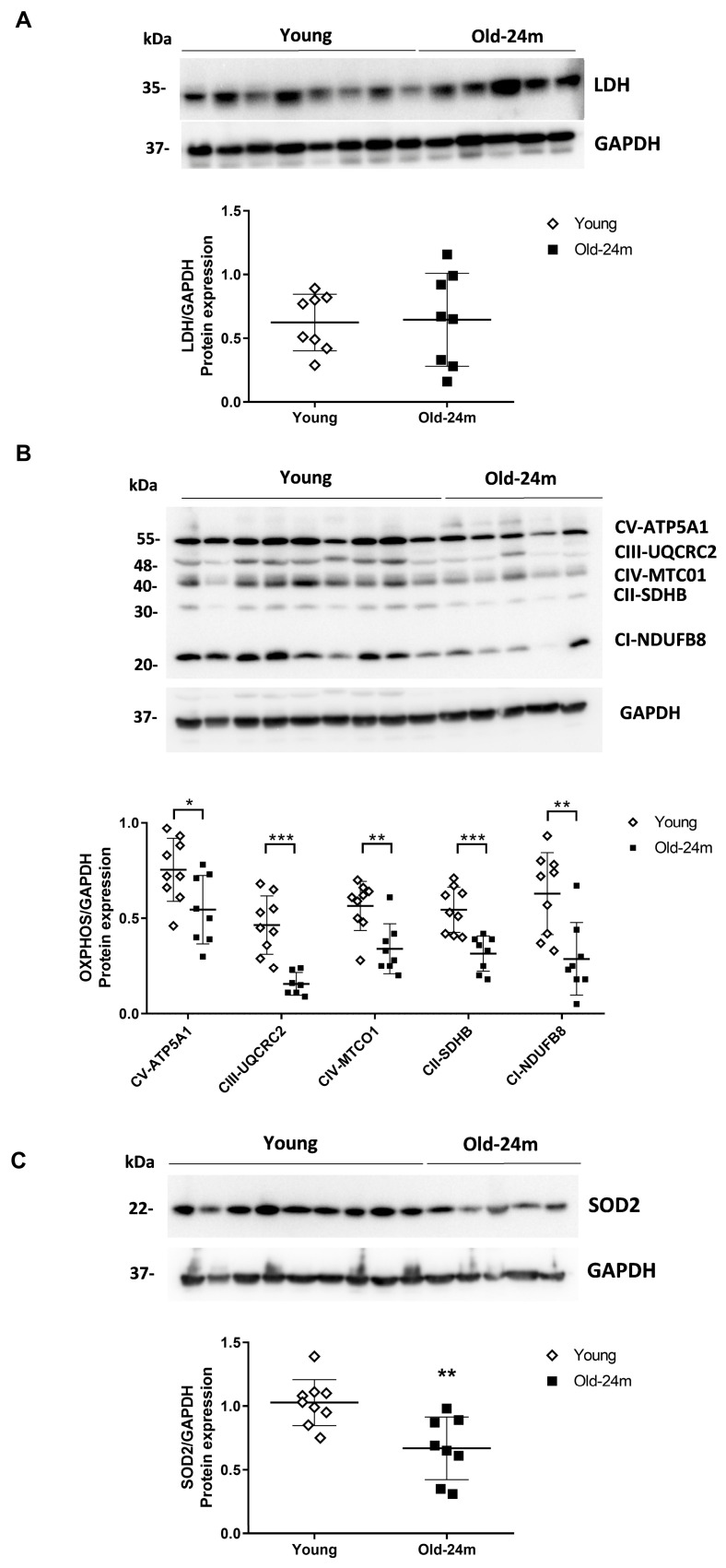
Aged mice skeletal muscle exhibits impairments in key proteins involved in mitochondrial function. Quadriceps muscle protein extracts isolated from young mice (5 months) and old mice (24 months) fed with standard diet were evaluated. (**A**) LDH, (**B**) OXPHOS subunits and (**C**) SOD2 protein expressions were analyzed by Western blot. A representative blot is shown in each case. Graphs represent values obtained from densitometric analysis of the bands from 9 young mice (Young) and 8 old mice (Old-24m). Densitometric values were normalized with endogenous GAPDH. Results are presented as individual data points for each animal with mean ± standard deviation (SD). Young (white diamond); Old-24m (black square). * *p* < 0.05; ** *p* < 0.01; and *** *p* < 0.001.

To determine whether the changes observed in cells due to high phosphate content also occur in muscle tissue, some in vivo studies were conducted in old mice (24 months) which present hyperphosphatemia compared to young mice (5 months) [14]. Considering the metabolism and redox balance alterations previously described above in cells, we studied some of these parameters in our animal model. First, we analyzed lactate dehydrogenase (LDH), the protein involved in production of lactate. LDH protein expression was measured in quadriceps samples of young and old mice, presenting no significant changes (Figure 5A). Secondly, the expression of ETC subunits was analyzed in order to clarify the effect of age-associated hyperphosphatemia on oxidative phosphorylation (Figure 5B). The proteins studied were ATP Synthase (CV-ATP5A1), Coenzyme Q (CIII-UQRC2), Mitochondrial cytochrome C oxidase subunit 1 (CIV-MTCO1), Succinate Dehydrogenase (CII-SDHB), and NADH Dehydrogenase (CI-NDUFB8). The results showed a significant decrease in each subunit protein expression in old mice compared to young animals (*p* = 0.0245; *p* =0.00018; *p* = 0.0028; *p* = 0.00051; and *p* = 0.00354). In addition, old skeletal muscles presented a significant reduction in SOD2 protein expression (*p* = 0. 0035), a relevant mitochondrial protein involved in redox regulation (Figure 5C). The expression of genes *Hk2*, *Pgk1* and *Eno1* were also examined, but no significant changes were observed (Supplementary Figure S3). These data show a dysregulation of oxidative metabolism in quadriceps of old mice and suggest that there is no compensatory increase in glycolysis factors.

### 2.6. Low-Phosphate Diet Enhances Expressions of LDH and Key Proteins for Mitochondrial Function in Aged Skeletal Muscle

**Figure 6:**
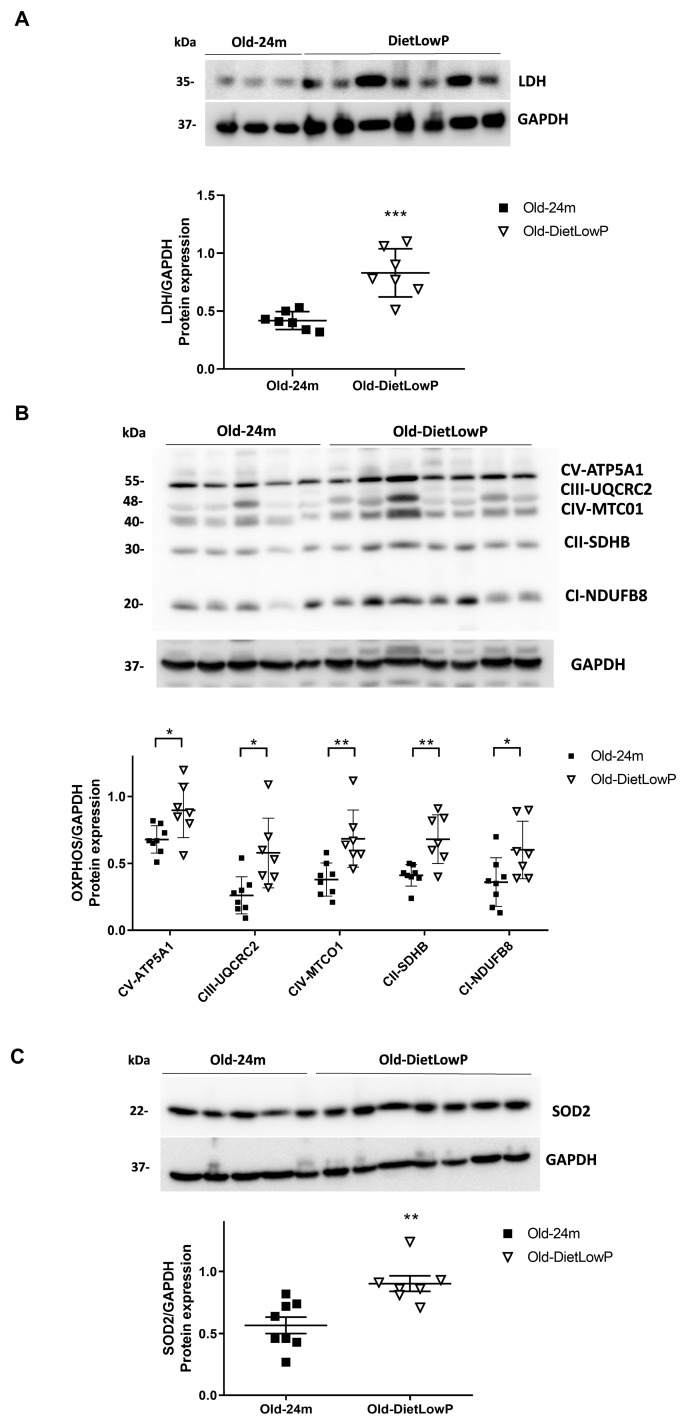
Dietary phosphate restriction increases the expression of LDH, OXPHOS and SOD2 in skeletal muscle of aged mice. Quadriceps muscle protein extracts were isolated from old mice (24 months) fed with standard diet and old mice (24 months) fed with a low-phosphate diet (Old-Diet LowP) for three months before sacrifice. (**A**) LDH, (**B**) OXPHOS subunits and (**C**) SOD2 protein expression were studied by Western blot. A representative blot in each case is shown. Graphs represent values obtained from densitometric analysis of the bands from 8 old mice (Old-24m) and 7 old mice fed with low-phosphate diet (Old-DietLowP). Densitometric values were normalized with endogenous GAPDH. Results are shown as individual data points for each animal with mean ± standard deviation (SD). Old-24m (Black square); Old-DietLowP (White inverted triangle). * *p* < 0.05; ** *p* < 0.01; and *** *p* < 0.001.

To establish an association between the observed changes in the measured parameters in old mice and phosphate levels, we studied quadriceps samples from old mice fed with a low-phosphate diet for three months. As we published recently, these animals have lower serum phosphate levels compared to old mice fed with standard diet [14]. Firstly, the expression of LDH was analyzed, revealing a significant increase in aged animals on a phosphate-restricted diet respect to those on a standard diet (Figure 6A) (*p* = 0. 0004). On the other hand, the previously mentioned OXPHOS subunits were also analyzed, and results showed an increase in the expression of each subunit in aged mice fed with a low-phosphate diet (Figure 6B) (*p* = 0.0194; = 0.0102; *p* = 0.0043; *p* = 0.0022; and *p* = 0.0347). Furthermore, an increase in the expression of SOD2 was also observed in animals fed with a hypophosphatemic diet (Figure 6C) (*p* = 0. 0031). Our results reveal an interesting relationship between hyperphosphatemia in old mice and alterations in proteins involved in glycolytic metabolism, oxidative phosphorylation and mitochondrial oxidative damage, which are recovered in old mice with dietary phosphate restriction.

### 2.7. Phosphate Binder Administration Increases the Expression of CIV-MTCO1 Subunit in Skeletal Muscle of Aged Mice

**Figure 7:**
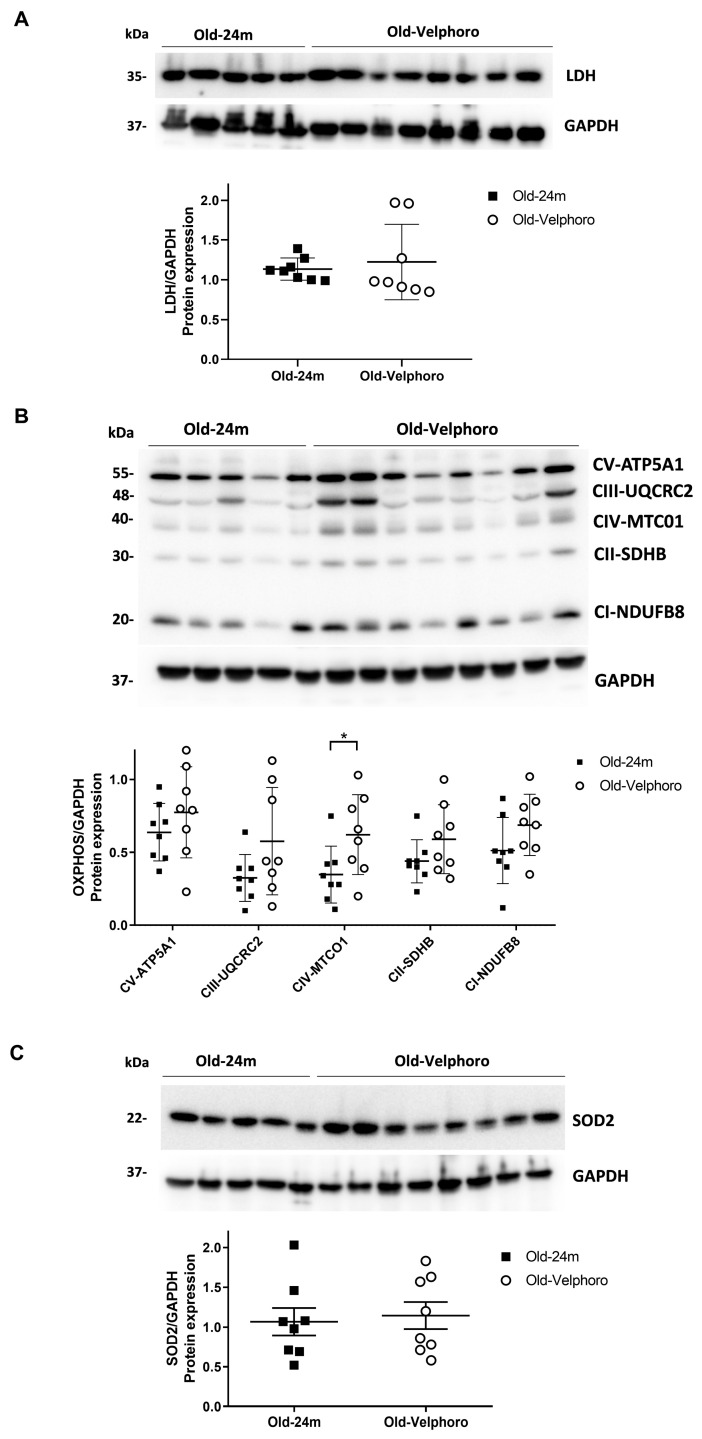
Phosphate binder administration increases the expression of CIV-MTCO1 subunit in skeletal muscle of aged mice. Quadriceps muscle protein extracts were isolated from old mice (24 months) fed with standard diet and from old mice (24 months) fed for the last three months of their life with a standard diet supplemented with the phosphate binder sucroferric oxyhydroxide powder (Velphoro^®^) (Old-Velphoro). (**A**) LDH, (**B**) OXPHOS subunits and (**C**) SOD2 protein expressions were evaluated by Western blot. A representative blot is shown in each case. Graphs represent values obtained from densitometric analysis of the bands from 8 old mice (Old-24m) and 8 old mice fed with Velphoro^®^ (Old-Velphoro). Individual data points for each animal are shown, with mean ± standard deviation (SD). Densitometric analysis was normalized with endogenous GAPDH. Old-24m (Black square); Old-Velphoro (White circle). * *p* < 0.05.

To reinforce the association between hyperphosphatemia and metabolic changes found in muscle from old animals, we analyzed quadriceps from a group of old mice fed with a phosphate binder. For this, a group of 24-month-old mice was fed for the last three months of their life with standard diet supplemented with 5% sucroferric oxyhydroxide powder (Velphoro^®^). This group, with a significant reduction in serum phosphate [14], was compared with old mice (24 months) fed with a standard diet which present hyperphosphatemia. Expression of LDH, OXPHOS subunits and SOD2 proteins were studied. In this case, the expression of LDH (Figure 7A), most of the OXPHOS subunits (Figure 7B) and SOD2 (Figure 7C) remained significantly unchanged. However, the expression of subunit CIV-MTCO1 was significantly increased in the Velphoro^®^ group when compared to control aged skeletal muscle (Figure 7B) (*p* = 0. 0372). These results suggest that the phosphate binder improved mitochondrial function, although it was not as effective as the hypophosphatemic diet.

To analyze the dose–response association between serum phosphate levels and mitochondrial respiratory chain proteins, a quadratic regression analysis was performed. Results shown in Supplementary Figure S4 reveal a consistent inverted U-shaped relationship between serum phosphate and all five respiratory chain proteins (β_2_ < 0 in all cases), with protein expression peaking at intermediate phosphate levels (vertex 12–17 mg/dL) and declining at higher concentrations, although the quadratic term did not reach statistical significance in any model. The F test was statistically significant for CIV-MTCO1 (*p* = 0.037) and CIII-UQCRC2 (*p* = 0.049), while CI-NDUFB8, CII-SDHB and CV-ATP5A1 displayed similar trends without reaching significance. Old-24m animals, characterized by the highest serum phosphorus levels, showed the lowest protein expression and clustered on the descending limb of the curve. In contrast, Young, Old-DietLowP and Old-Velphoro groups exhibited intermediate phosphorus levels with higher mitochondrial protein expression. Collectively, these results support an association between age-related hyperphosphatemia and reduced expression of mitochondrial respiratory chain proteins, particularly in complexes III and IV.

### 2.8. The Degree of Sarcopenia Is Related to Low Expression of Several Key Proteins Involved in Mitochondrial Function and Health

**Figure 8:**
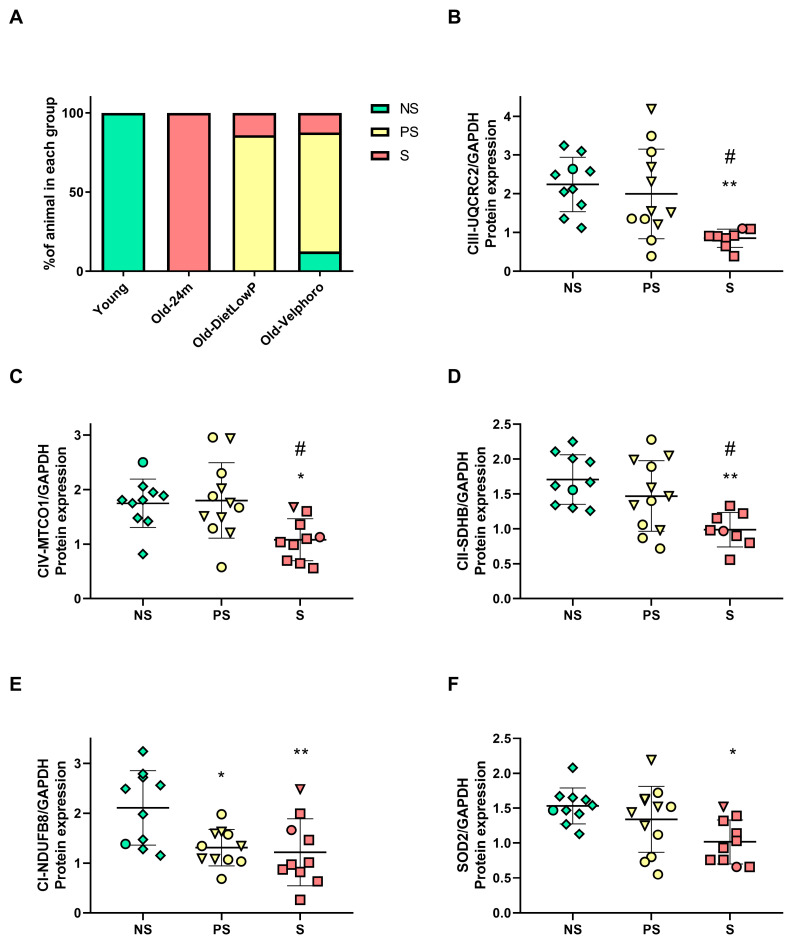
Association between skeletal muscle mitochondrial protein expression and the degree of sarcopenia. Experimental groups: 5-month-old mice (young); 24-month-old mice fed with standard diet (Old-24m); 24-month-old mice fed with low-phosphate diet (Old-DietLowP); and 24-month-old mice with standard diet and Velphoro^®^ (Old-Velphoro). (**A**) Graph with color-coded representation of percentage of animals stratified using signs of sarcopenia. Graphs of (**B**) CIII-UQRC2, (**C**) CIV-MTCO1, (**D**) CII-SDHB, (**E**) CI-NDUFN8 and (**F**) SOD2 within each stratification level. Protein expression was analyzed by Western blot and normalized to GAPDH. Data are presented as individual points for each animal with mean ± standard deviation (SD). Color meaning: non-sarcopenic (NS; green); possible sarcopenic (PS; yellow); and sarcopenic (S; red). Shape meaning: diamond (young); square (Old-24m); inverted triangle (Old-Diet); and circle (Old-Velphoro). * *p* < 0.05, ** *p* < 0.01 vs. NS; and # *p* < 0.05 vs. PS.

We previously reported that aged animals fed a low-phosphate diet or Velphoro^®^ showed improvements in muscle strength, muscle mass, and physical performance compared with age-matched animals fed the standard diet. A direct link between serum phosphate levels and sarcopenia signs was well established [14]. In order to evaluate the potential association between the observed metabolic alterations and sarcopenia, we conducted multiple analyses. Firstly, animals used in the present study were stratified, according to the three parameters previously mentioned, into three degrees: non-sarcopenic (NS), possible sarcopenic (PS) and sarcopenic (S) (data from grip strength; gastrocnemius mass and transition time used for stratification are in Supplementary Table S1) Consequently, Figure 8A shows that 100% of individuals in the young group were non-sarcopenic and mice in the 24-month-old group were all sarcopenic; meanwhile, only 14.3% and 12.5% of the animals in the Old-DietLowP and Old-Velphoro groups, respectively, presented sarcopenia. Old animals fed with a low-phosphate diet showed a reduction from S to PS in 85.7% of cases. Velphoro^®^ administration reduced S by 87.5% (75% of PS and 12.5% of NS). Serum phosphate levels from the animal cohort used in this work were 15.97 ± 3.03 mg/dL in the young animals; 22.6 ± 4.6 mg/dL in 24-month-old mice; 17.9 ± 2.4 mg/dL in the phosphate diet restriction group; and 12.3 ± 1.07 mg/dL in the Velphoro^®^ administration group.

Secondly, the relationship between sarcopenia degree and some of the proteins previously studied in the four experimental groups was analyzed. The expression of the CIII-UQRC2, CIV-MTCO1 and CII-SDHB subunits presented a significant decrease in sarcopenic animals when compared to NS and PS (Figure 8B (** *p* = 0. 0051; # *p* = 0. 0173), Figure 8C (* *p* = 0. 0245; # *p* = 0. 0106), and Figure 8D (** *p* = 0. 0023; # *p* = 0. 0368). Expression of CI-NDUFB8 showed a significant reduction in PS and S degrees when compared to NS (* *p* = 0. 0156; ** *p* = 0. 008) (Figure 8E). Finally, SOD2 expression was diminished in sarcopenic mice with respect to the NS (*p = 0. 0108) (Figure 8F). Expressions of the ATP Synthase subunit and LDH were analyzed in the same manner, with no changes observed (data provided in Supplementary Figure S5).

Altoghether, the results suggest a strong association between altered expression of mitochondrial proteins and the degree of sarcopenia development.

## 3. Discussion

Sarcopenia is a multifactorial condition, with age-related hyperphosphatemia identified as one of the contributing factors. A key feature in the development of sarcopenia is the progressive loss of skeletal muscle regenerative capacity. Sarcopenic muscle is characterized by impaired myogenic differentiation together with a shift toward increased fibrogenic and adipogenic differentiation [4,13].

To specifically address the contribution of hyperphosphatemia to these alterations, we employed an in vitro experimental model based on mouse myoblasts (C2C12) induced to differentiate in the presence of horse serum, a well-established and widely used system for studying myogenic differentiation [16]. Using this model, we investigated the isolated effect of hyperphosphatemia on myogenic differentiation. Previously, we have demonstrated that hyperphosphatemia impairs C2C12 myogenic differentiation [13] contributing to the appearance of sarcopenia signs in old mice [14]. In the current study, we used C2C12 cells differentiated in the presence of a high concentration of extracellular phosphate donor as a model of hyperphosphatemic in vitro condition in order to deeply examine the mechanism involved in this effect. Our findings show that myotubes differentiated in the presence of high levels of phosphate present mitochondrial dysfunction and reprogram their metabolism inducing a shift towards glycolysis. Furthermore, in our old mice experimental model, we showed an association between mitochondrial impairment and a higher presence of serum phosphate levels, correlating with an advanced stage of sarcopenia.

Myoblasts rely primarily on glycolytic energy production [15]. During myogenic differentiation, some relevant physiological changes occur, including a shift from glycolysis to an increased oxidative phosphorylation, enhanced ATP production and elevated mitochondrial content, among others [17]. In this work, we demonstrate that this process is altered in C2C12 differentiated under hyperphosphatemic conditions. While upregulation of a key glycolytic protein such as GLUT1 is observed, oxidative phosphorylation is simultaneously reduced, as indicated by decreased CI-NDUFB8 expression (subunit of complex I in ETC), resulting in reduced ATP production. These findings could be related to previous studies on how high phosphate intake in the diet reduces ATP production in skeletal muscle [18]. Although glycolysis can sustain high rates of ATP production, its lower ATP yield per glucose molecule limits its capacity to compensate for reduced oxidative phosphorylation [19]. In mice, high-phosphate diets induce metabolic gene changes reflecting increased glycolysis and reduced fatty acid oxidation, probably as compensation for the impaired oxidative metabolism [20]. Nevertheless, this adaptation does not fully support muscle energetic demands, as evidenced by impaired function, reduced exercise capacity, and muscle atrophy [20,21].

To ensure that elevated extracellular phosphate during myogenic differentiation induced this metabolic change, we studied some other genes related to glycolysis. We observed that, after treating C2C12 cells during the differentiation process with high phosphate levels, there was a significant increase in the expression of genes such as *Hk2*, *Pgk1* or *LdhA* coupled by higher lactate production. HK2 catalyzes the first step of glycolysis [22], afterwards PGK1 generates ATP through substrate-level phosphorylation [23] and, finally, LDHA reduces pyruvate to lactate, the main product of glycolysis in skeletal muscle [24]. As a loading control, we used GAPDH in these experiments, despite its role as a glycolytic enzyme. However, we verified that its expression remained stable under our experimental conditions. Alterations in oxidative phosphorylation limit mitochondrial oxidative capacity, promoting glycolytic flux and increasing lactate production in muscle cells, a process that contributes to metabolic compensation [24,25]; however, as mentioned above, glycolysis cannot fully compensate for the reduced production of ATP [22].

To further explore the mechanism involved in these alterations, different parameters related to mitochondrial function were studied. C2C12 cells differentiated in the presence of high levels of phosphate presented a significant reduction in citrate synthase (CS) activity, the limiting enzyme of the tricarboxylic acid cycle (TCA) and therefore a fundamental regulator of energy production through mitochondrial respiration [26]. In addition, a reduced expression of the CI-NDUFB8 was also observed in the differentiation process under hyperphosphatemic conditions. This protein is an essential structural subunit of mitochondrial complex I, whose activity represents the primary entry point of electrons into the respiratory chain and a rate-limiting step for oxidative phosphorylation, thus playing a central role in mitochondrial energy production [27,28]. Furthermore, a decrease in cytochrome C oxidase activity was observed despite the lack of changes in COXIV-4 protein expression. Reduced expression of complex I and decreased activity of complex IV suggest that impairments in individual complexes may be functionally connected. In relation to this, it is known that mitochondrial complexes I and IV assemble into higher-order structures known as supercomplexes or respirasomes [29]. This organization creates structural interdependence among the individual complexes and supports efficient electron transfer and oxidative phosphorylation [30], a principle likely relevant in skeletal muscle mitochondria. Additionally, measured levels of mitochondrial ROS were increased during myogenic differentiation in the presence of high levels of phosphate, and no changes were observed in mRNA expression of the SOD2 enzyme. This could indicate an inability to protect against oxidative damage caused by excess phosphate. On the other hand, when this ROS production was decreased in cells treated with NAC and BGP, GLUT1 protein expression did not increase and mRNA *Hk2* expression did not change, compared with cells differentiated only in the presence of BGP, suggesting a distinct regulatory mechanism on glycolytic proteins or genes by phosphate-induced ROS. This point will be the aim for further experiments to elucidate the exact mechanism involved.

Our findings indicate that metabolic change and mitochondrial dysfunction occur in cells during myogenic differentiation under hyperphosphatemic conditions. Metabolic switching towards lactate production contributes to maintaining cellular energy when mitochondrial function is impaired [31]. Reduced CS activity can limit Krebs cycle flux, decrease NADH production, and alter the NAD^+^/NADH balance, compromising electron transport chain activity [32]. These observations are consistent with age-related mitochondrial dysfunction and altered muscle metabolism reported in mouse models [33], supporting the idea that decreased Krebs cycle activity contributes to impaired energy metabolism.

To explore the mechanism underlying the effect of hyperphosphatemia on mitochondria, we analyzed mitochondrial health and dynamics. Mitochondrial content in treated differentiated myotubes were studied and no changes were observed. However, hyperphosphatemia produced a reduction in the mitochondrial membrane potential, which reflects the proton gradient driving ATP production and indicates impaired mitochondrial integrity and compromised bioenergetic activity [34]. Moreover, regarding our results of mitochondrial dynamics, an excess of phosphate during myogenic differentiation produces a decrease in the expression of fission protein DRP1, together with an increase in fusion protein MFN2, indicating a clear imbalance between fusion–fission that dysregulate mitochondria function. Ageing skeletal muscle displays alterations in mitochondrial dynamics; however, the direction of these changes remains unclear [35]. Several studies report a reduction in MFN2 expression with ageing, suggesting a shift toward increased mitochondrial fission and an association with sarcopenia [36]. In contrast, other evidence highlights an opposite remodeling, showing an increased fusion–fission index, reflected by a higher MFN2/DRP1 ratio in aged skeletal muscle [37]. Together, these findings indicate that mitochondrial fission–fusion balance is altered during ageing but with inconsistent outcomes across studies. On the other hand, proper mitochondrial function in skeletal muscle requires a balance between biogenesis and mitophagy [35]. Our results show increased TFAM, elevated BNIP3 expression, and a higher LC3-II/LC3-I ratio, reflecting enhanced autophagosome formation. In ageing muscle, dysregulation of mitochondrial dynamics and quality control occurs alongside mitochondrial dysfunction and increased oxidative stress, while mitophagy-related signaling is activated despite an overall impairment in autophagic flux [37,38,39].

To determine the relevance in vivo of these changes, we carried out studies in quadriceps muscles isolated from old mice (24 months old) compared with young mice (5 months old). Previously, we have shown that older animals in this cohort displayed hyperphosphatemia and signs of sarcopenia when fed the standard diet compared to the young group. However, when old mice were fed with a hypophosphatemic diet or a phosphate binder (Velphoro^®^) for three months before being sacrificed, serum phosphate was reduced and some of these signs were prevented in aged animals [14].

In this work, we analyzed some proteins related to glycolytic or oxidative metabolism in quadriceps from this animal model. We found that LDH protein expression from young and old mice remained unchanged. Some studies report an increase in anaerobic glycolysis and lactate accumulation in ageing muscles [40], while others observe reductions in the activities of glycolytic enzymes and intermediates [41]. No changes in our study may be explained by age-related changes in muscle fiber composition. Fast-twitch glycolytic fibers show higher LDH activity, but ageing is associated with a relative loss of these fibers and an increase in type I oxidative fibers, which could compensate for any expected upregulation of LDH in aged muscle [42,43,44].

However, our results present a significant age-related decrease in the expression of various OXPHOS subunits and SOD2. Consistently, several studies have demonstrated that ageing is associated with a decrease in the mitochondrial oxidative phosphorylation capacity in skeletal muscle, in addition to a reduction in the activity of key OXPHOS components [45]. Also, decreased mitochondrial respiratory efficiency, oxidative stress, lower ATP production, and a reduction in mitochondrial protein content, mitochondrial DNA abundance, and transcriptional output in older individuals have been reported [46,47,48].

To demonstrate the involvement of hyperphosphatemia in these changes, we performed studies on the quadriceps of old mice fed with a hypophosphatemic diet for three months. Our results showed that aged mice fed with the low-phosphate diet exhibited increased LDH protein expression compared to their counterparts on a standard diet. This observation could be related to an increase in the percentage of glycolytic fibers observed in this group of mice in previous studies [14] and a greater LDH activity in glycolytic muscle fibers [49]. Additionally, a noticeable increase in the protein expression of the five OXPHOS subunits studied and the enzyme SOD2 was observed in old mice fed with a phosphate-restricted diet compared with those fed with a standard diet. Clearly, these results show a noteworthy role of hyperphosphatemia in metabolic changes in the skeletal muscle of old mice. In this sense, it has been shown that excessive phosphate intake alters metabolic pathways and compromises the energy supply necessary for muscle performance [50], and the overexpression of SOD2 in mice confers protection against mitochondrial oxidative stress by preserving ATP production during ageing [51].

Additionally, a group of old mice were fed with the phosphate binder Velphoro^®^ during the last three months of life as a practical alternative to dietary phosphate restriction. The same proteins were analyzed, and in this case only a beneficial effect in the CIV-MTCO1 subunit protein expression was found compared to old mice fed with a standard diet. These results suggest that Velphoro^®^ is less effective than the hypophosphatemic diet at preventing alterations in OXPHOS protein expression in aged skeletal muscle. We previously reported that Velphoro^®^ supplementation results in a more pronounced reduction in serum phosphate levels than the hypophosphatemic diet [14]. To analyze the association between the dose of serum phosphate and the expression of mitochondrial respiratory chain proteins, we perform a quadratic regression model. The consistently negative quadratic coefficients observed across all respiratory chain proteins suggest a non-linear, dose-dependent association between serum phosphate levels and these proteins, whereby the optimal protein expression is reached at intermediate phosphorus concentrations (estimated vertex 12–17 mg/dL), moderate reduced expression at lower concentration and stronger reduction at higher phosphorus levels. Old-24m mice in our study clustered at the highest phosphorus concentrations and exhibited the lowest expression of mitochondrial proteins. Importantly, dietary phosphate restriction and pharmacological intervention with the phosphate binder Velphoro^®^ partially shifted serum phosphorus concentrations toward intermediate values, coinciding with moderate improvements in mitochondrial protein expression. Overall, our data reinforce the notion that maintaining serum phosphate within an optimal physiological window is associated with the preservation of OXPHOS expression and energy metabolism in ageing skeletal muscle.

To complete this study, mice were stratified according to their degree of sarcopenia, which is directly associated with hyperphosphatemia. The stratification was obtained based on the measurement of strength, muscle mass and physical performance of the animals in this study, according with the protocol previously published [52]. Old mice showed decreased strength and physical performance and hyperphosphatemia with respect to the young mice. Feeding a hypophosphatemic diet or administering Velphoro^®^ significantly improved these parameters. Data from the stratification proved that both low-phosphate-diet and Velphoro^®^ administration significantly reduced the degree of sarcopenia, linking high phosphate levels with greater disease development. Additionally, proteins that showed a reduced expression in sarcopenic mice were CIII-UQRC2, CIV-MTCO1, CII-SDHB, CI-NDUFB8 and SOD2. After applying these stratification criteria, we performed a comparative analysis of the expression levels of several mitochondrial proteins in mice classified as non-sarcopenic (NS), possible sarcopenic (PS), and sarcopenic (S). This analysis shows that sarcopenic mice exhibit markedly reduced mitochondrial protein expression compared with both NS and PS groups, suggesting that sarcopenia severity may be associated with a progressive decline in mitochondrial function.

In summary, our findings demonstrate that age-related hyperphosphatemia is associated with perturbed metabolic homeostasis and compromises mitochondrial function and quality in skeletal muscle and that these changes scale with the severity of sarcopenia. Notably, dietary phosphate restriction protects skeletal muscle from mitochondrial and metabolic alterations, and partially reversed sarcopenic features provoked by age-related phosphate excess. Altogether, these results contribute to a better understanding of the mechanisms by which age-related hyperphosphatemia contributes to skeletal muscle deterioration and the development of sarcopenia, establishing an association between elevated phosphate levels and metabolic and mitochondrial dysfunction. Moreover, they support the notion that the reduction in phosphate levels may constitute a beneficial strategy to prevent or reduce the development of sarcopenia. Additional studies will be necessary to elucidate the intracellular mechanisms through which high levels of phosphate impair mitochondrial function, as well as to elucidate the resulting physiological consequences on metabolism in the in vivo experimental models. In addition, further studies will be conducted in female models to determine whether there are sex-specific differences.

## 4. Materials and Methods

### 4.1. Cell Culture and In Vitro Experimental Design

C2C12 myoblast (CRL-1772) cell line was obtained from the American Type Culture Collection (ATCC) (Manassas, VA, USA). Myoblasts were cultured in 60 mm dishes, 35 mm dishes or 24-well plates with Dulbecco’s Modified Eagle Media (DMEM) with 4.5 g/L glucose from Corning (10-013-CV) (Sigma-Aldrich, St. Louis, MO, USA). Then, 35 mm culture dishes were coated with 0.2% gelatin to facilitate cell adhesion to the glass surface. We added 10% of fetal bovine serum (Sigma-Aldrich, St. Louis, MO, USA) and 100 μg/mL streptomycin and 100 U/mL penicillin (Thermo Fisher Scientific, Waltham, MA, USA). After 24 h of proliferation, myoblasts were cultured in DMEM with 2% horse serum (HS) for 168 h to induce myogenic differentiation in the presence or absence of 10 mM of β-glycerophosphate (BGP) (35675, Merck-Millipore, Burlington, MA, USA) as the phosphate donor. For experiments with the ROS scavenger, N-acetylcysteine, NAC (A9165, Sigma-Aldrich, Merck Life Science, Madrid, Spain) was added at 100μM to cells for 16 h each day before refreshing the cellular medium in the absence or presence of BGP during myogenic differentiation for 168 h. Cells were used at passages 3–10 and were grown at 37 °C in an atmosphere with 5% of CO_2_.

### 4.2. Animal Studies

Male C57BL/6J mice from Janvier Laboratories were divided into four experimental groups, young mice (5 months old) fed with milled standard diet (Tekland global 14% protein, from ENVIGO, Indianapolis, IN, USA; containing 0.6% of phosphate); old mice (24 months) fed with the milled standard diet; old mice fed with milled hypophosphatemic diet containing 0.2% of phosphate (EF Low Phosphate S9723-E020, Ssniff-Spezialdiäten GmbH, Soest, Germany) during the last three months of life; and old mice fed with milled standard diet supplemented with 5% of phosphate binder (sucroferric oxyhydroxide powder: Velphoro^®^, Vifor Pharma, Zurich, Switzerland) for the last three months of life, to analyze the effect of a low-phosphate diet or of a phosphate binder. This experiment was performed in only one cohort of 32 mice. Animals were randomly placed in cages with an average of three animals per cage and equipped with environmental enrichment items. The animals were maintained in pathogen-free rooms under standard conditions of light (12:12 h light–dark cycle) and temperature (~24 °C). Animals received water and food ad libitum.

One week before their respective sacrifices (5 or 24 months of age), relevant tests were conducted to measure strength and physical performance, as explained in previous publications [14]. In addition, at the moment of euthanasia, the mice were anesthetized and blood was obtained by cardiac puncture to determine serum phosphate levels. Following this, gastrocnemius muscle was extracted and weighted to determine muscle mass (normalized to body weight) [14]. Finally, quadriceps muscles were extracted and stored in RNA-later (AM7021, Invitrogen, Thermo Fisher Scientifc, Waltham, MA, USA) at −80 °C for protein expression determinations.

The Ethics Committee from the University of Alcalá and the Comunidad de Madrid (protocol code PROEX 210/17 and 255.7/21) approved the animal study protocol. Experimental protocols and study design were performed according to the Guide for the Care and Use of Laboratory Animals published by the US National Institute of Health (Publication No. 85-23, revised 1996), European Union and National regulations (EU Directive 2010/63/EU, Spanish State law 3/2007 on animal care and the Royal Decrees RD1201/2005 and RD53/2013 on the protection of experimental animals and other scientific purposes).

### 4.3. Stratification of Sarcopenic Degree

Severe sarcopenia is diagnosed when diminished strength, low muscle mass/quality, and impaired physical performance occur together. Using clinically relevant criteria based on the European Working Group on Sarcopenia in Older People (EWGSOP) [53], the entire cohort of animals was stratified into three different sarcopenic degrees: non-sarcopenic (NS), possible sarcopenic (PS) and sarcopenic (S). The three criteria used were the results of the grip test (strength), the gastrocnemius mass/body weight (muscle quantity/quality) and the transition time from the static rod bar test (physical performance). To define a positive marker of sarcopenia, we used two standard deviations below (strength and muscle mass) or above (transition time) the mean of the young group in each criterion [52]. The sarcopenic degree of all mice was obtained summing positive sarcopenic markers of all tests: NS (zero positive markers); PS (one positive marker); and S (two or more positive markers). To allow for comparison between the protein expression of the 3 degrees of sarcopenia, the values for each mouse were normalized relative to the mean for old mice within the same blot.

### 4.4. Western Blot Analysis

Proteins from C2C12 cells and quadriceps samples were obtained using a lysis buffer that contains 10 mM Tris-HCl pH 7.5, 1 mM EDTA, 1% Triton X-100, and 0.1% sodium deoxycholate, supplemented with protease and phosphatase inhibitors (04693116001/04906837001, Roche Diagnostics S.L., Barcelona, Spain). Protein concentrations were determined by the Bradford method using a Bio-Rad protein assay kit (Richmond, CA, USA). An equal concentration of samples (10–20 µg) was separated on 9–12% SDS-polyacrylamide gels and transferred onto PVDF membranes. The molecular weight marker used was the BlueStar Plus Protein Marker (523272, Corning, Düren, Germany), with a molecular weight range of 10–240 kDa. Blots were blocked with 5% of non-fat dry milk and incubated with primary and secondary antibodies. The primary antibodies used were GLUT1 (ab115730, Abcam, Cambridge, UK); OXPHOS Cocktail (MS604-300, Abcam, Cambridge, UK); COX-IV Subunit 4 (11242-1-AP, Proteintech, Planegg-Martinsried, Germany); DRP1 (ab184248, Abcam, Cambridge, UK); MFN2 (12186-1-AP, Proteintech, Planegg-Martinsried, Germany); LC3B-I/LC3B-II (3868S, Cell Signalling, Danvers, MA, USA); LDH (EP1566y, Abcam, Cambridge, UK); SOD2 (13141, Cell Signalling, Danvers, MA, USA); Tubulin (T4026, Sigma-Aldrich, St. Louis, MO, USA); and GAPDH (G8795, Sigma-Aldrich, St. Louis, MO, USA). Secondary antibodies used were against rabbit (AP132P, Merck-Millipore, Burlington, MA, USA) or mouse (A4416, Sigma-Aldrich, St. Louis, MO, USA). Immunoblots were detected by chemiluminescence (3217487, Pierce ECL Western Blotting Substrate; Thermo Fisher Scientific, MA, USA) and images were obtained using the ImageQuant LAS 500 System (General Electric Healthcare, Chicago, IL, USA). Densitometries were analyzed by using ImageJ software v1.53t (National Institutes of Health, Bethesda, MD, USA).

### 4.5. RNA Extraction and Quantitative RT-qPCR

RNA from cells and quadriceps samples was extracted with Nyzol reagent according to the manufacturer’s protocol (MB18502, Nzytech, Lisbon, Portugal). cDNA was obtained by transcribing equal amounts of RNA with a High-Capacity cDNA RT Kit (4368814, Applied Biosystems, Thermo Fisher Scientific, Waltham, MA, USA). Quantitative PCRs were performed in QUANTSTUDIO™ 12K FLEX software version 1.3 (Applied Biosystems Inc., Foster City, CA, USA) and were analyzed using the ΔΔCt method. The Taqman primers used were *Hk2* (Mm00443385_m1), *Sod2* (Mm01313000_m1) and *Gapdh* (Mm99999915_g1). SYBR-green primers were as follows:

*Pgk1* (GenBank: NM_008828.3; product length: 120 bp; Fw: AACCTCCGCTTTCATGTAGAG, Rv: GACATCTCCTAGTTTGGACAGTG);

*Eno1* (GenBank: NM_001379127.2; product length: 350 bp; Fw: AACCCTGAAGTCATCCTGCCTGTC, Rv: TTGCCAGACCTGTAGAACTCGGAG); *LdhA* (GenBank: NM_010699.2; product length: 135 bp; Fw: AGGCTCCCCAGAACAAGATT, Rv: TCTCGCCCTTGAGTTTGTCT);

*Tfam* (GenBank: NM_009360.4; product length: 122 bp; Fw: AAGGGAATGGGAAAGGTAGAG, Rv: ACAGGACATGGAAAGCAGATTA);

*Bnip3* (GenBank: NM_009760.4; product length: 403 bp; Fw: TGAATCTGGACGAAGTAGCTCC, Rv: CAGACGCCTTCCAATGTAGATC);

*Gapdh* (GenBank: NM_001411844.1; product length: 173 bp; Fw: CCACCCAGAAGACTGTGGAT, Rv: CACATTGGGGGTAGGAACAC).

### 4.6. ATP Measurement

ATP determination was assessed by using the ATP Detection Assay Kit (700410, Cayman Chemical, Ann Arbor, MI, USA) in cells differentiated in the presence or absence of 10 mM of BGP for 168 h. Cell lysate from 24-well plates with 100% confluent was processed, and total nmoles of ATP were obtained by luminescence assay measurement, following the manufacture’s protocol using a Victor X4 Multilabel Plate Reader (PerkinElmer, Waltham, MA, USA).

### 4.7. Extracellular Lactate Measurement

Extracellular L-lactate content was determined by using the L-Lactate Assay Kit (700510-96, Cayman Chemical, MI, USA). The day before the 168 h of differentiation were completed, the culture medium was changed to measure L-lactate production during the last 24 h. The culture medium from plates with 1 × 10^6^ cells was recollected and centrifuged at 2000× *g* for 10 min at 4 °C. Following the manufacturer’s instructions, measurements were taken using a colorimetric assay at 535 nm of wavelength using a Victor X4 Multilabel Plate Reader (PerkinElmer, Waltham, MA, USA). Measures were normalized with µg of protein obtained with the Bradford method.

### 4.8. Citrate Synthase and Cytochrome C Oxidase Activities

Citrate synthase and cytochrome C oxidase activity were evaluated by using a Citrate synthase activity Kit (ab239712, Abcam, Cambridge, UK) and Cytochrome C Oxidase Assay Kit (ab239711, Abcam, Cambridge, UK), respectively. Cell lysates from myoblast differentiated in the presence or absence of 10 mM of BGP for 168 h were used for the colorimetric assays performed at wavelengths of 412 nm and 550 nm, respectively, following the manufacturer’s protocols. Activity measures were normalized using the protein concentration and were performed using a FLUOstar Omega Plate Reader v5.70 (BMG Labtech, Ortenberg, Germany).

### 4.9. Analysis of Mitochondrial Content, Membrane Potential and Oxidative Stress

Myoblasts were differentiated for 168 h in the presence or absence of 10 mM BGP in 35 mm dishes. After treatment, all determinations were performed on living cells using a Leica SP5 confocal microscope (Leica Microsystems, Wetzlar, Germany) or Fluorescence Inverted Microscope DMi8 Leica (Leica Microsystems, Wetzlar, Germany). Mitochondrial mass was determined by using Mitotracker^TM^ Red FM dye (M22425, Invitrogen, Thermo Fisher, Waltham, MA, USA) in an excitation wavelength range of 581⁄644. To determine the mitochondrial membrane potential, a probe based on Tetramethylrhodamine methyl ester or TMRM (I34361, Invitrogen, Thermo Fisher Scientifc, Waltham, MA, USA) was used in an absorption/emission range of 548/574 nm. Finally, mitochondrial superoxide production was determined using MitoSOX^TM^ Red (M36008, Invitrogen, Thermo Fisher, Waltham, MA, USA), with a wavelength range of ∼396/610 nm. All probes were used according to the manufacturer’s instructions. After incubation with these probes, the cell nuclei were stained with Hoechst in blue (H3570, Invitrogen, Thermo Fisher, Waltham, MA, USA) for 10 min before being analyzed under the microscope. Images were analyzed with ImageJ software v1.53t (National Institutes of Health, Bethesda, MD, USA). A total of three fields were analyzed for each condition in each of the different experiments, quantifying the fluorescence intensity present in the myotubes for each of the probes and the results were normalized by the myotube nuclei content, stained with Hoechst.

### 4.10. Statistical Analysis

All statistical analyses were performed in Graphpad Prism 8 (San Diego, CA, USA). The number of repetitions per experiment was at least three. Results were represented as mean ± standard error of mean (SEM) for cell-based experiments or as mean ± standard deviation (SD) for animal experiments. For cases in which the effect of treatment and time were studied simultaneously, a two-way ANOVA test was used. For all other cases, statistical analyses were performed using paired or unpaired tests, depending on the experimental design. For independent samples, Student’s *t* test was used, or the Mann–Whitney U test when data did not follow a normal distribution. For paired data, the paired *t* test or Wilcoxon signed-rank test were applied, as appropriate based on normality. For comparisons involving more than two groups, one-way ANOVA was performed, followed by appropriate post hoc tests when necessary. Differences were considered statistically significant when presenting at least a probability of less than 5% (*p* < 0.05).

The dose–response relationships between the serum phosphorus levels and OXPHOS protein expression in quadriceps from 32 mice distributed across four experimental groups (Young, Old-24m, Old-DietLowP and Old-Velphoro) were assessed by quadratic regression analysis (y = β_0_ + β_1_x + β_2_x^2^), with the statistical significance of the quadratic term (β_2_) and overall model fit (F-test) reported for each protein.

## Data Availability

The original contributions presented in this study are included in the article/Supplementary Materials. Further inquiries can be directed to the corresponding author.

## Supplementary Materials

The following supporting information can be downloaded at https://www.mdpi.com/article/10.3390/ijms27135662/s1.
