# Supplementary material for: Age-Related Hyperphosphatemia Is Associated with Metabolic and Mitochondrial Alterations During Myogenic Differentiation and in Skeletal Muscle from Old Mice
Source: Int J Mol Sci. 2026 Jun 23;27(13):5662. doi: 10.3390/ijms27135662 (PMC13361694; doi:10.3390/ijms27135662)
Supplement: Supplementary file 1 [file ijms-27-05662-s001.zip › Suplementary Material Figure S1.pdf]

Age-Related Hyperphosphatemia is associated with Metabolic and Mitochondrial Alterations during Myogenic Differentiation and in Skeletal Muscle from Old Mice

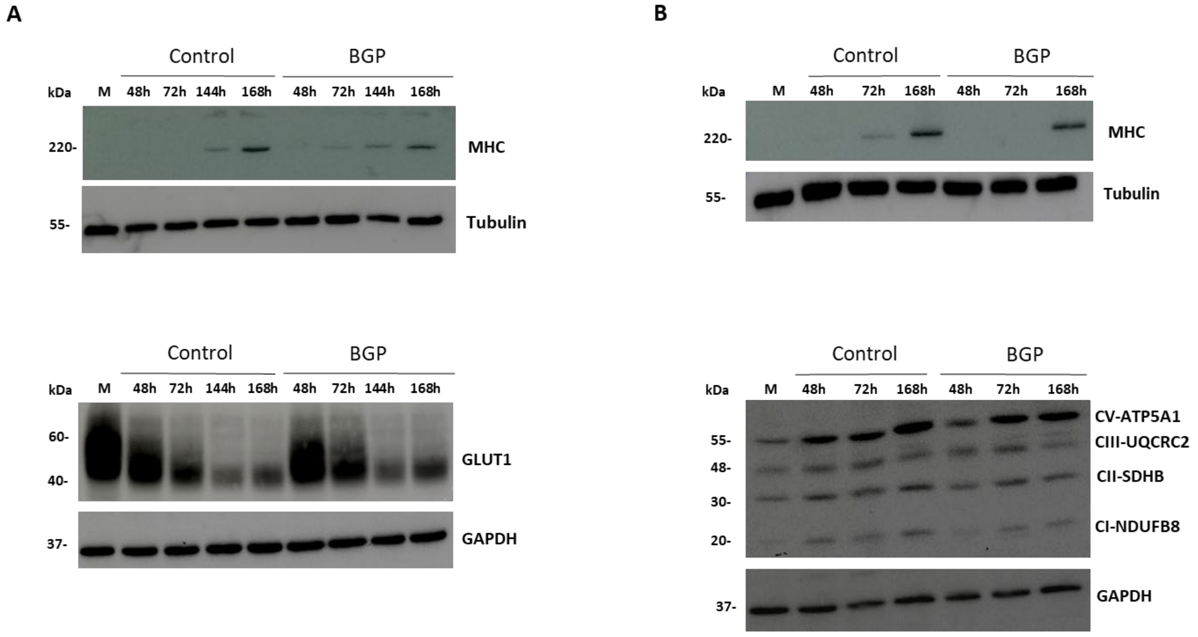

Figure S1: Hyperphosphatemia reduces Myosin Heavy Chain (MHC) protein expression in differentiating C2C12 cells. C2C12 cells were analyzed at undifferentiated myoblasts (M), 48 hours, 72 hours, 144 hours and 168 hours of differentiation in absence (Control) or presence of 10 mM β-glycerophosphate (BGP) during differentiation. **(A)** Representative immunoblots of MHC, GLUT1 and their respective endogenous controls. **(B)** Representative immunoblots of MHC and OXPHOS complexes and their respective endogenous controls.
