# Supplementary material for: Age-Related Hyperphosphatemia Is Associated with Metabolic and Mitochondrial Alterations During Myogenic Differentiation and in Skeletal Muscle from Old Mice
Source: Int J Mol Sci. 2026 Jun 23;27(13):5662. doi: 10.3390/ijms27135662 (PMC13361694; doi:10.3390/ijms27135662)
Supplement: Supplementary file 1 [file ijms-27-05662-s001.zip › Suplementary Material Figure S2.pdf]

Age-Related Hyperphosphatemia is associated with Metabolic and Mitochondrial Alterations during Myogenic Differentiation and in Skeletal Muscle from Old Mice

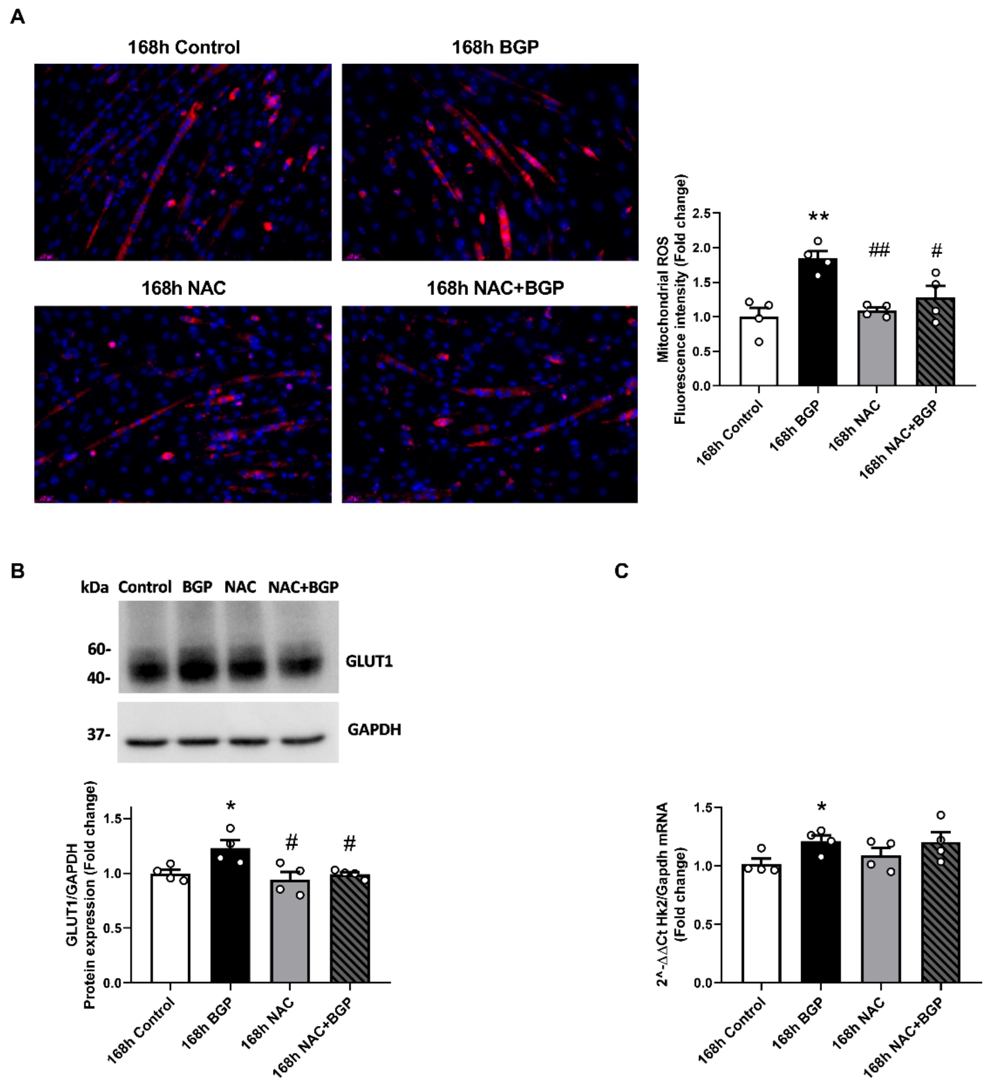

Figure S2: Hyperphosphatemia-induced oxidative stress modulates GLUT1 protein expression, without affecting *Hk2* gene expression, in differentiated C2C12 cells. C2C12 cells were analyzed after 168 hours of differentiation in the absence (Control) or presence of 10 mM β-glycerophosphate (BGP) and/or 100 μM N-acetylcysteine (NAC) during differentiation. **(A)** Mitochondrial ROS production was determined by fluorescence microscopy using superoxide mitochondrial probe (MitoSOX™ red fluorescence), and normalized by nuclei of myotubes content (Hoechst in blue). Representative images obtained at 40× magnification are shown. Scale bars: 50μM. **(B)** GLUT1 protein expression was determined by Western blot. Densitometries were normalized to endogenous GAPDH. **(C)** mRNA expression of *Hk2* was measured using RT-qPCR and normalized to *Gapdh*. Results are expressed as individual data points relative to the mean of 168h Control and are presented as the mean ± SEM from 4 independent experiments. * $p < 0.05$ vs 168h Control; ** $p < 0.01$ vs 168h Control; # $p < 0.05$ vs 168h BGP; ## $p < 0.01$ vs 168h BGP.
