# Supplementary material for: Age-Related Hyperphosphatemia Is Associated with Metabolic and Mitochondrial Alterations During Myogenic Differentiation and in Skeletal Muscle from Old Mice
Source: Int J Mol Sci. 2026 Jun 23;27(13):5662. doi: 10.3390/ijms27135662 (PMC13361694; doi:10.3390/ijms27135662)
Supplement: Supplementary file 1 [file ijms-27-05662-s001.zip › Suplementary Material Figure S3.pdf]

Age-Related Hyperphosphatemia is associated with Metabolic and Mitochondrial Alterations during Myogenic Differentiation and in Skeletal Muscle from Old Mice

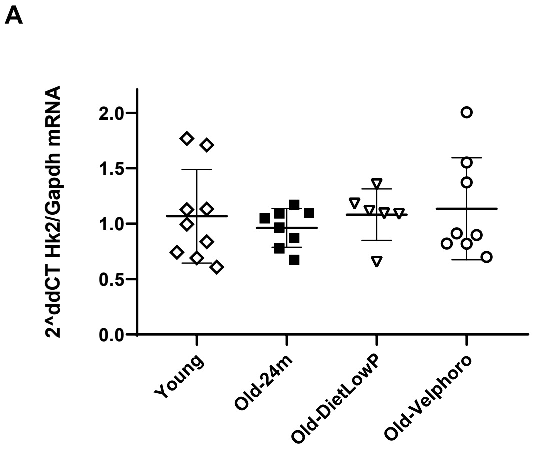

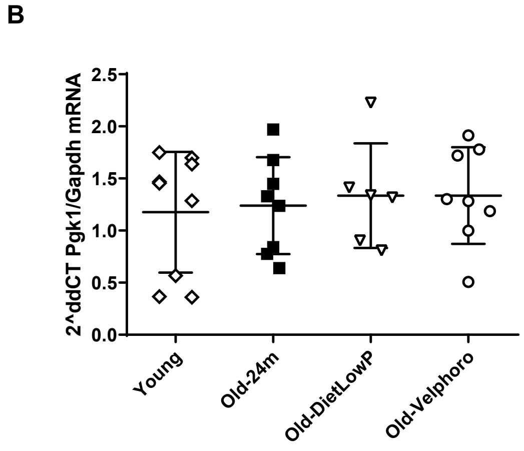

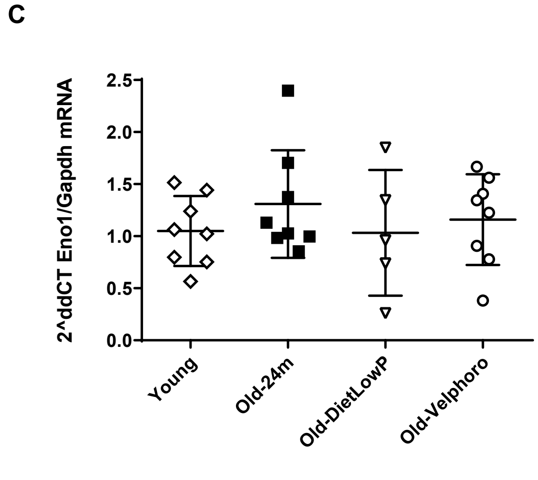

Figure S3: No significant differences in glycolytic gene expression in quadriceps muscle. Experimental groups: 5-month-old mice (Young); 24-month-old mice fed with standard diet (Old-24m); 24-month-old mice fed with a low-phosphate diet for the last three months of their life (Old-DietLowP); 24-month-old mice fed with standard diet supplemented with the phosphate binder sucroferric oxyhydroxide powder (Velphoro®) for the last three months of their life (Old-Velphoro). Graphs of **(A)** *Hk2*, **(B)** *Pgk1* and **(C)** *Eno1* mRNA expression. Gene expression was analyzed by RT-qPCR and normalized to endogenous *Gapdh*. Data are presented as individual points for each animal with mean ± standard deviation (SD). Shape meaning: White diamond (Young); Black square (Old-24m); White inverted triangle (Old-Diet); White circle (Old-Velphoro). No significant differences were detected between groups.
