# Supplementary material for: Age-Related Hyperphosphatemia Is Associated with Metabolic and Mitochondrial Alterations During Myogenic Differentiation and in Skeletal Muscle from Old Mice
Source: Int J Mol Sci. 2026 Jun 23;27(13):5662. doi: 10.3390/ijms27135662 (PMC13361694; doi:10.3390/ijms27135662)
Supplement: Supplementary file 1 [file ijms-27-05662-s001.zip › Suplementary Material Figure S4.pdf]

Age-Related Hyperphosphatemia is associated with Metabolic and Mitochondrial Alterations during Myogenic Differentiation and in Skeletal Muscle from Old Mice

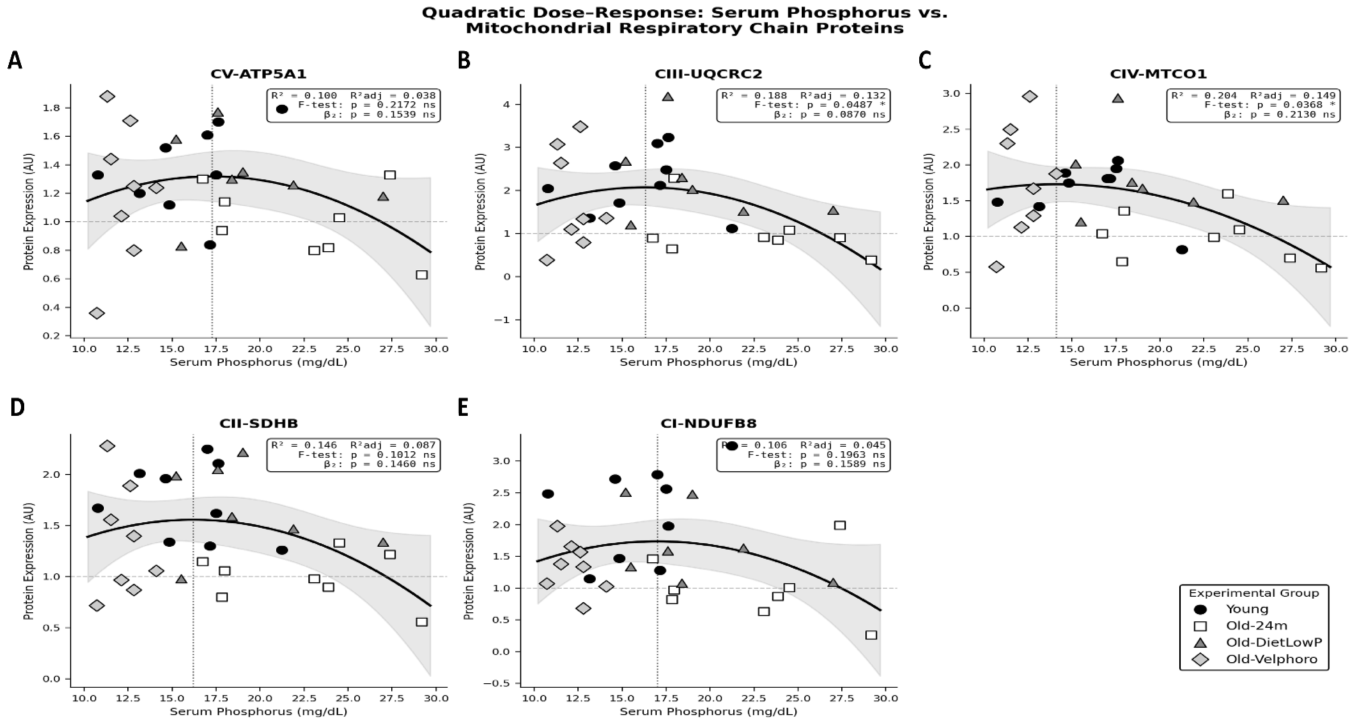

Figure S4. Association between serum phosphate and mitochondrial respiratory chain proteins. Experimental groups: 5-month-old mice (Young); 24-month-old mice fed a standard diet (Old-24m); 24-month-old mice fed a low-phosphate diet (Old-DietLowP); and 24-month-old mice fed a standard diet supplemented with Velphoro® (Old-Velphoro). The quadratic regression models were used to assess the dose-response relationship between serum phosphate levels (mg/dL) and protein expression of **(A)** CV-ATP5A1, **(B)** CIII-UQCRC2, **(C)** CIV-MTCO1, **(D)** CII-SDHB, and **(E)** CI-NDUFB8. Data are presented as individual values for each animal. Symbol legend: circle (Young), square (Old-24m), triangle (Old-DietLowP), diamond (Old-Velphoro). Statistical significance was defined as $p < 0.05$.
