# Supplementary material for: Age-Related Hyperphosphatemia Is Associated with Metabolic and Mitochondrial Alterations During Myogenic Differentiation and in Skeletal Muscle from Old Mice
Source: Int J Mol Sci. 2026 Jun 23;27(13):5662. doi: 10.3390/ijms27135662 (PMC13361694; doi:10.3390/ijms27135662)
Supplement: Supplementary file 1 [file ijms-27-05662-s001.zip › Suplementary Material Figure S5.pdf]

Age-Related Hyperphosphatemia is associated with Metabolic and Mitochondrial Alterations during Myogenic Differentiation and in Skeletal Muscle from Old Mice

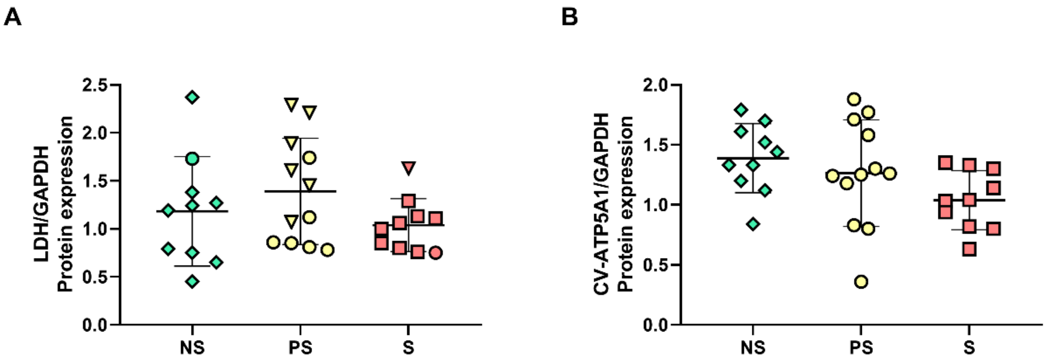

Figure S5. No association between LDH and ATP5A1 protein expression and the degree of sarcopenia in skeletal muscle. Experimental groups: 5-month-old mice (Young); 24-month-old mice fed a standard diet (Old-24m); 24-month-old mice fed a low-phosphate diet (Old-DietLowP); and 24-month-old mice fed a standard diet supplemented with Velphoro® (Old-Velphoro). Graphs show **(A)** LDH and **(B)** CV-ATP5A1 levels within each stratification category. Data are presented as individual values for each animal with mean ± standard deviation (SD). Colour code: Non-Sarcopenic (NS, green); Possible Sarcopenic (PS, yellow); Sarcopenic (S, red). Symbol code: Diamond (Young); Square (Old-24m); Inverted triangle (Old-DietLowP); Circle (Old-Velphoro). No statistically significant differences were observed among sarcopenia categories.
