# Supplementary material for: Age-Related Hyperphosphatemia Is Associated with Metabolic and Mitochondrial Alterations During Myogenic Differentiation and in Skeletal Muscle from Old Mice
Source: Int J Mol Sci. 2026 Jun 23;27(13):5662. doi: 10.3390/ijms27135662 (PMC13361694; doi:10.3390/ijms27135662)
Supplement: Supplementary file 1 [file ijms-27-05662-s001.zip › Suplementary Material Table S1.pdf]

Age-Related Hyperphosphatemia is associated with Metabolic and Mitochondrial Alterations during Myogenic Differentiation and in Skeletal Muscle from Old Mice

	Young n=9	Old-24m n=8	Old-DietLowP n=7	Old-Velphoro n=8
Grip strength (gf/gr body weight)	2.52 ± 0.32	1.52 ± 0.25 * (* $p < 0.0001$)	2.19 ± 0.22 # (# $p < 0.0001$)	2.18 ± 0.19 * # (* $p = 0.0488$; # $p < 0.0001$)
Gastrocnemius mass/body mass (mg/g)	3.87 ± 0.54	3.49 ± 0.32	3.71 ± 0.38	3.26 ± 0.57
Transition time (s)	2.80 ± 0.4	7.58 ± 1.95 * (* $p = 0.0002$)	6.37 ± 1.62 * (* $p < 0.0001$)	4.69 ± 0.95 * # $ (* $p< 0.0001$; # $p = 0.0035$; $ $p = 0.0266$)

Table S1. Measurements of strength, muscle mass and physical performance in experimental groups. Experimental groups: Five-month-old mice (Young); 24-month-old mice fed with standard diet (Old-24m); 24-month-old mice fed with a low-phosphate diet for the last three months of their life (Old-DietLowP); 24-month-old mice fed with standard diet supplemented with the phosphate binder sucroferric oxyhydroxide powder (Velphoro®) for the last three months of their life (Old-Velphoro). Values are presented as mean ± standard deviation (SD) for Grip strength (gf/gr body weight), Gastrocnemius mass/body mass (mg/g), and Transition time (s). Statistical significance is indicated as follows: * vs. Young, # vs Old-24m, $ vs Old-DietLowP ($p \leq 0.05$).
