# Supplementary figures and images for: Age-Related Hyperphosphatemia Is Associated with Metabolic and Mitochondrial Alterations During Myogenic Differentiation and in Skeletal Muscle from Old Mice
Source: Int J Mol Sci. 2026 Jun 23;27(13):5662. doi: 10.3390/ijms27135662 (PMC13361694; doi:10.3390/ijms27135662)

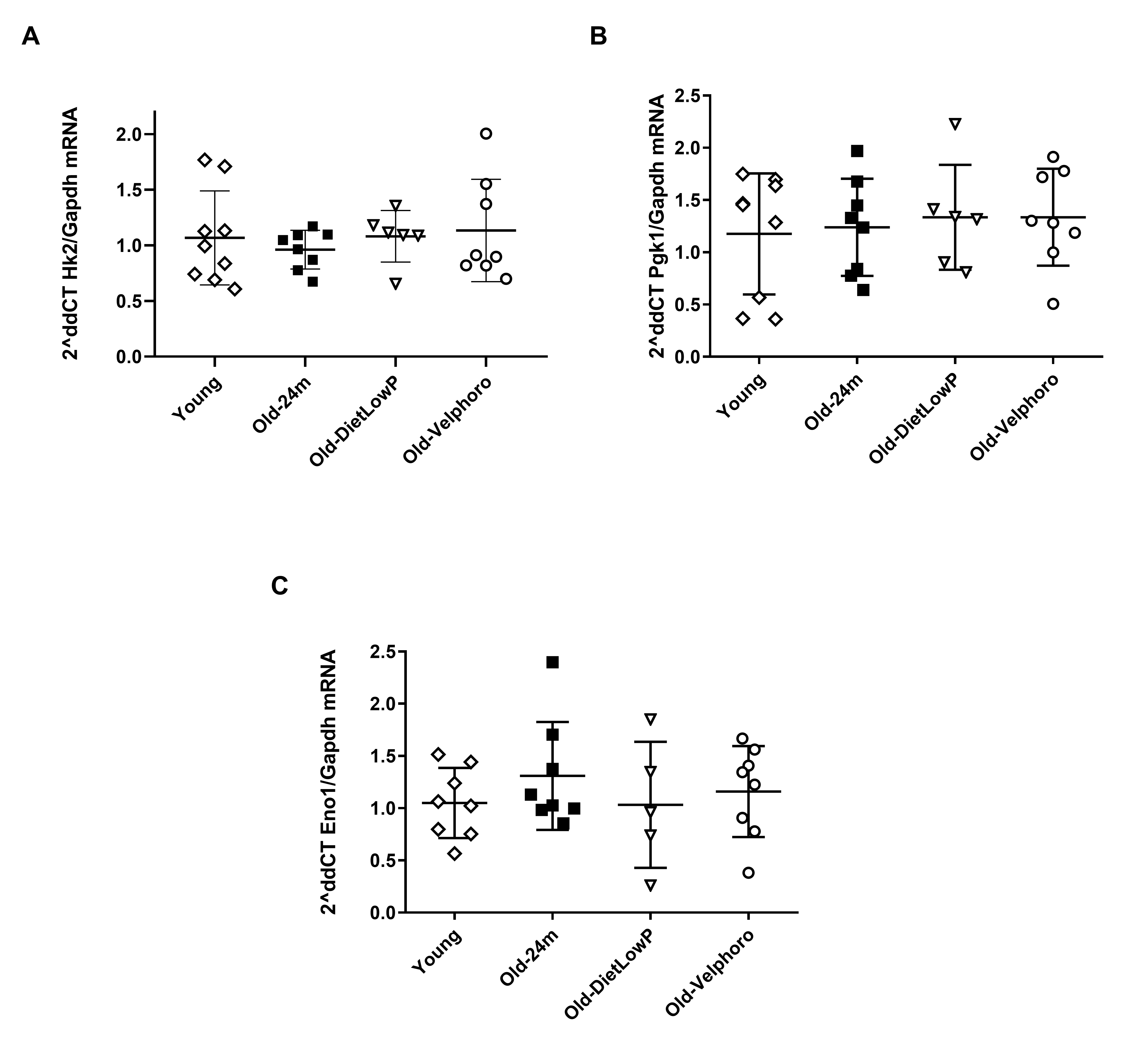

Supplement: Supplementary file 1 [file ijms-27-05662-s001.zip › Figure S3.tif]

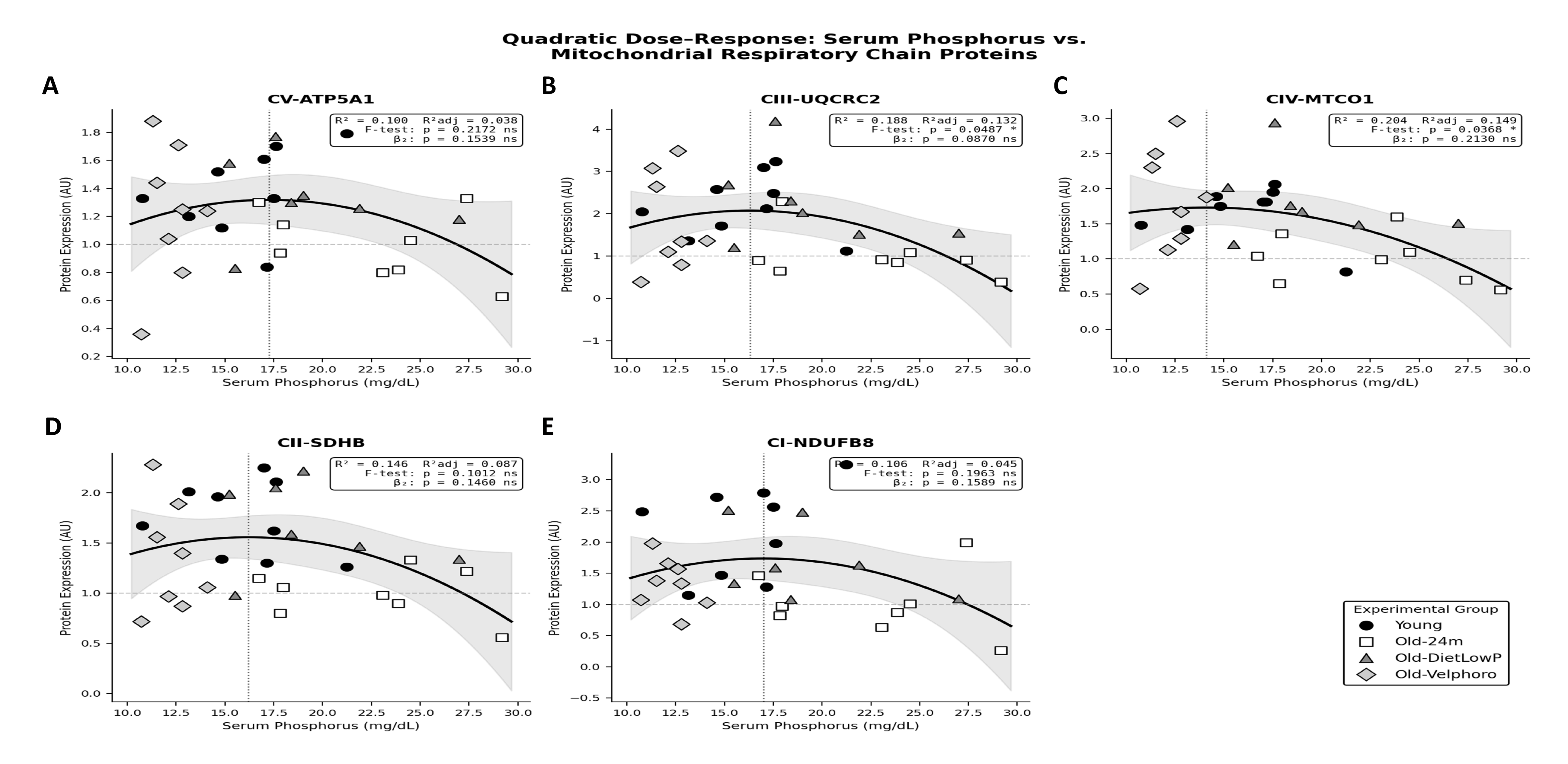

Supplement: Supplementary file 1 [file ijms-27-05662-s001.zip › Figure S4.tif]

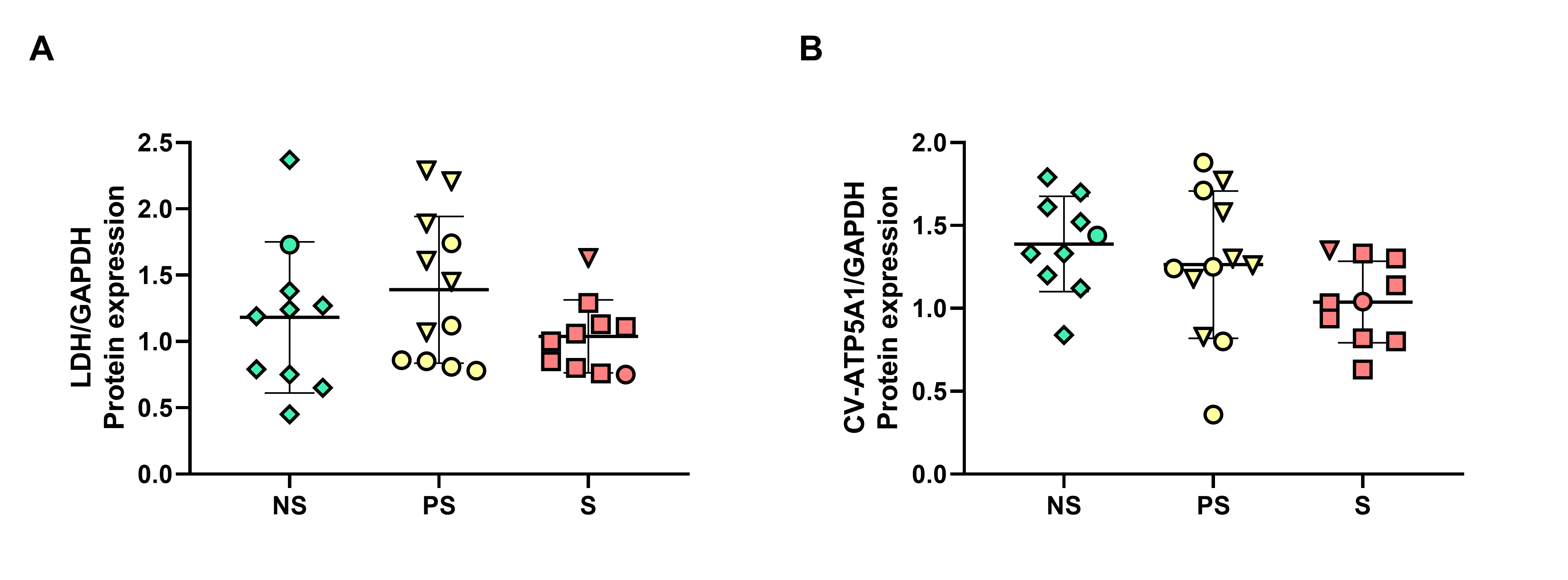

Supplement: Supplementary file 1 [file ijms-27-05662-s001.zip › Figure S5.tif]

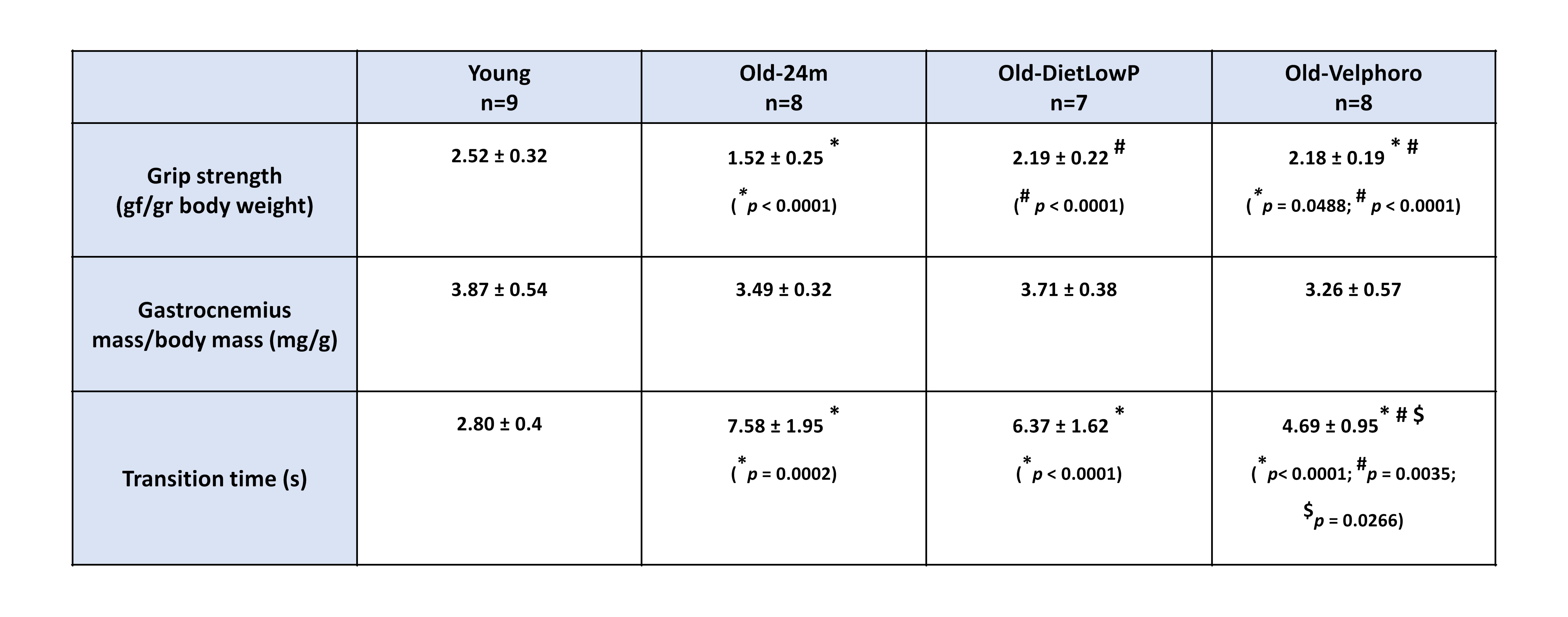

Supplement: Supplementary file 1 [file ijms-27-05662-s001.zip › Table S1.tif]

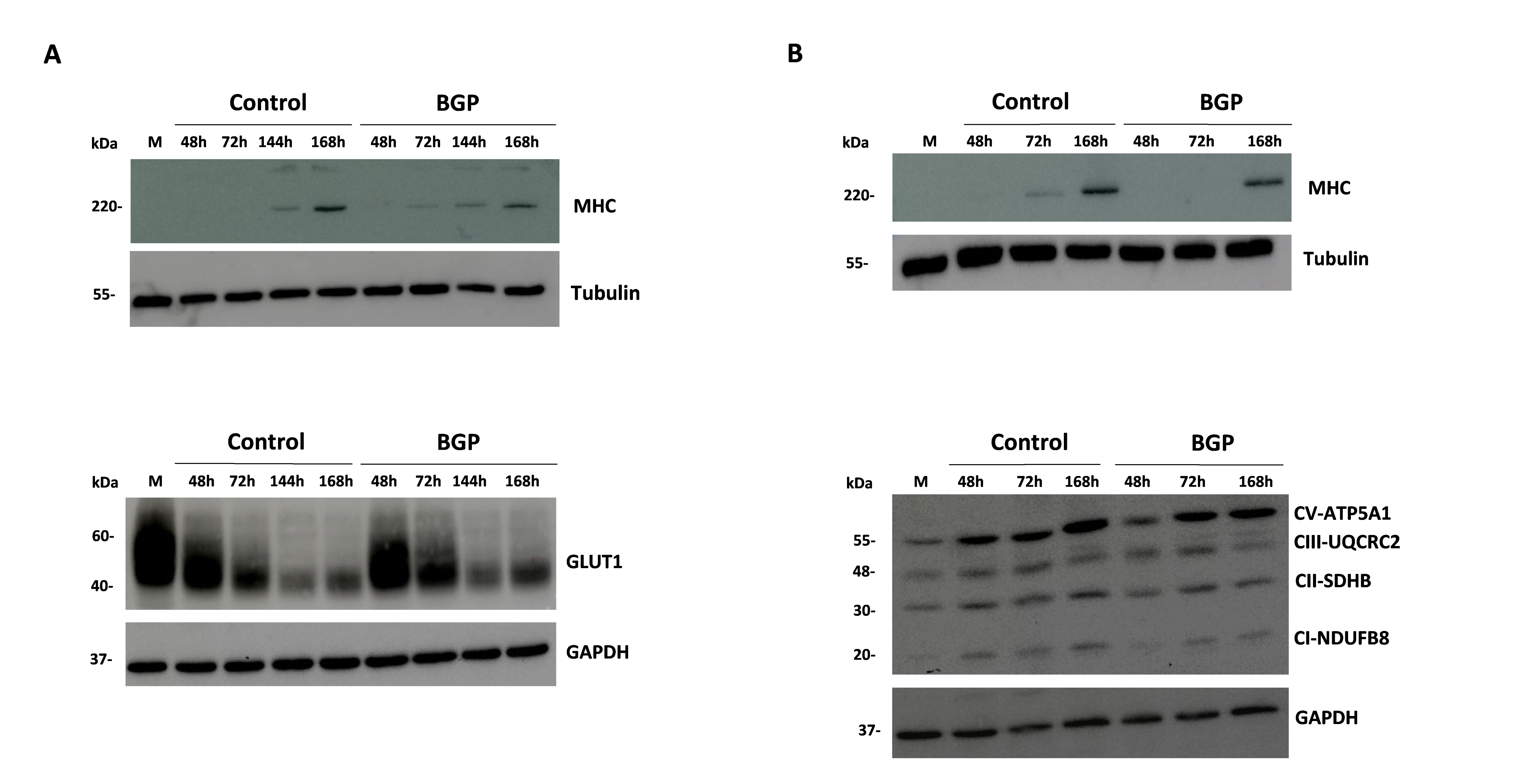

Supplement: Supplementary file 1 [file ijms-27-05662-s001.zip › Figure S1.tif]

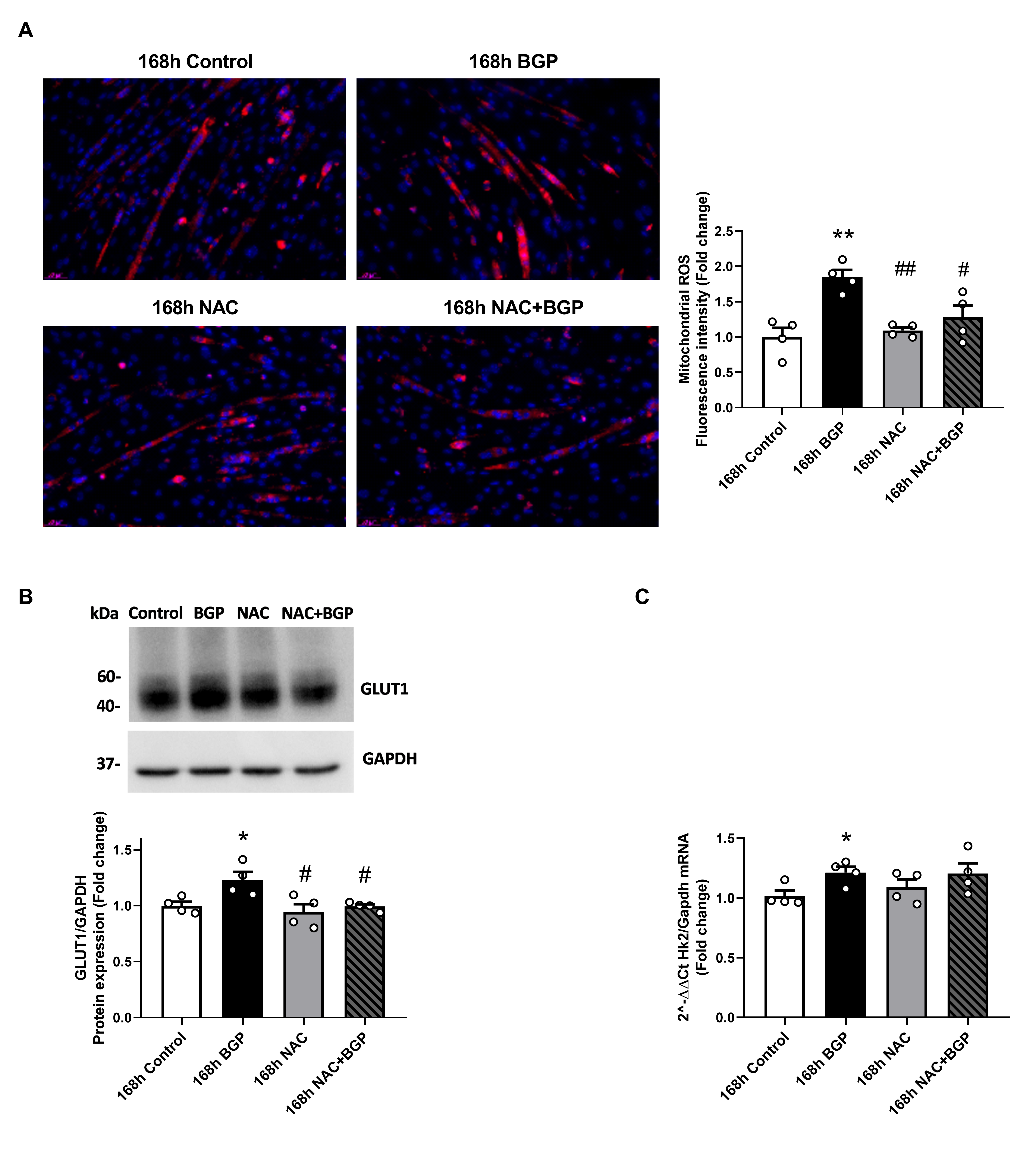

Supplement: Supplementary file 1 [file ijms-27-05662-s001.zip › Figure S2.tif]
